# Assessment of the Nutritional Status of Four Selected Rural Communities in KwaZulu-Natal, South Africa

**DOI:** 10.3390/nu13092920

**Published:** 2021-08-24

**Authors:** Laurencia Govender, Kirthee Pillay, Muthulisi Siwela, Albert Thembinkosi Modi, Tafadzwanashe Mabhaudhi

**Affiliations:** 1 Dietetics and Human Nutrition, School of Agricultural, Earth and Environmental Sciences, University of KwaZulu-Natal, Private Bag X01, Scottsville 3209, Pietermaritzburg 3201, South Africa; pillayk@ukzn.ac.za (K.P.); siwelam@ukzn.ac.za (M.S.); 2 Centre for Transformative Agricultural and Food Systems, School of Agricultural, Earth and Environmental Sciences, University of KwaZulu-Natal, Private Bag X01, Scottsville 3209, Pietermaritzburg 3201, South Africa; modiat@ukzn.ac.za (A.T.M.); mabhaudhi@ukzn.ac.za (T.M.)

**Keywords:** anthropometry, dietary assessment, malnutrition, nutritional assessment

## Abstract

Under- and over-nutrition co-exist as the double burden of malnutrition that poses a public health concern in countries of the developing regions, including South Africa (SA). Vulnerable groups such as pregnant women and children under five years are the most affected by malnutrition, especially in rural areas. Major contributing factors of malnutrition include food and nutrition insecurity, poverty, and unhealthy lifestyles. The current study aimed to assess the nutritional status, using selected anthropometric indices and dietary intake methods (repeated 24 h recall and food frequency), of four rural communities in KwaZulu-Natal (SA). Purposive sampling generated a sample of 50 households each in three rural areas: Swayimane, Tugela Ferry, and Umbumbulu and 21 households at Fountain Hill Estate. The Estimated Average Requirement cut-point method was used to assess the prevalence of inadequate nutrient intake. Stunting (30.8%; n = 12) and overweight (15.4%; n = 6) were prevalent in children under five years, whilst obesity was highly prevalent among adult females (39.1%; n = 81), especially those aged 16–35 years. There was a high intake of carbohydrates and a low intake of fibre and micronutrients, including vitamin A, thus, confirming the need for a food-based approach to address malnutrition and micronutrient deficiencies, particularly vitamin A deficiency.

## 1. Introduction

The double burden of malnutrition remains an ongoing challenge worldwide [1], especially in children [2]. The 2020 Global Nutrition Report indicated that stunting and wasting affected 149.0 and 49.5 million children under five years, respectively, while 40.1 million children under the age of five years were overweight [3]. In Africa, the number of stunted children under five years of age increased by 8.1 million between 2000 and 2017 [4]. Child malnutrition is most prevalent in the sub-Saharan African region, and there are inter-and-intra-country variations in malnutrition trends [1,2].

Several national studies have investigated the nutritional status of the South African population. The South African National Health and Nutrition Examination Survey (SANHANES-1) conducted in 2012 found that underweight (5.5%) and wasting (2.5%) rates had improved in children [5]. On the contrary, the prevalence of stunting among children increased by 5.4% between 2012 and 2016, indicating that stunting has worsened [5,6,7].

Earlier national studies on vitamin A deficiency (VAD) conducted in South Africa (SA) indicated a worsening trend; however, the 2012 SANHANES-1 study reported that the prevalence of VAD in children was still high, despite a 20% drop since 2005 [5,7,8,9]. The 2005 National Food Consumption Survey-Fortification Baseline (NFCS-FB) study showed that in children, the intake of energy and several essential micronutrients including, calcium, iron, selenium, vitamins D, C and E, riboflavin, niacin, vitamin B_6_, and folic acid, was less than two-thirds of the recommended dietary allowance (RDA) [7].

Undernutrition remains a concern among South African children and leads to several health conditions, including those linked to protein-energy malnutrition and micronutrient deficiencies [5,10,11,12]. The prevalence of undernutrition is attributed to poverty, food, and nutrition insecurity, inadequate infrastructure and poor access to health care facilities and limited education [13,14,15,16,17]. On the other hand, overweight and obesity are serious health concerns, especially in children, as it increases the risk of other chronic diseases of lifestyle later in life, such as cardiovascular disease (CVD), hypertension, and diabetes mellitus (DM) [18,19]. Childhood obesity, which is a form of over-nutrition, has several possible causes. These include increased consumption of poor-quality high-energy foods, overweight or obese parents, poor physical activity, and metabolic disorders [18]. In addition, stunting in children increases the risk of overweight and obesity in adulthood [2].

The 2005 NFCS-FB study found that one in ten South African children were overweight [20]. Similarly, a study conducted by Armstrong et al. (2006) found that the prevalence of overweight and obesity increased with age among African girls aged 6–13 years [21]. Likewise, a national study conducted in 2012 in SA reported that the prevalence of overweight and obesity was lower in boys (11.5% and 4.7%, respectively) than girls (16.5% and 7.1%, respectively). These results were consistent for all age groups. KwaZulu-Natal (KZN) was one of the provinces in SA with the highest rates of obesity among children with 6.1% and 8.5% among boys and girls, respectively [5]. Furthermore, the 2016 South African Demographic and Health Survey (SADHS) indicated that 13% of children under five were overweight [5,6]. These studies reiterate that over-nutrition is also a serious health concern in children [5,6,20,21]. From the national studies, it is evident that both under- and over-nutrition co-exist among South African children. However, data on the nutritional status of different population groups in rural and urban areas in KZN are limited. The national studies were conducted in specific districts and sub-districts within KZN, and therefore do not necessarily reflect the nutritional status of the KZN population as a whole. This further emphasizes a knowledge gap concerning the nutritional status of rural population groups in local municipalities.

Overnutrition is also prevalent among South African adults, especially women, where, according to the 2016 SADHS, one in five women were severely obese [6]. In addition, the 2012 SANHANES-1 study revealed that the prevalence of overweight and obesity was higher among women (24.8% and 39.2%, respectively) than men (20.1% and 10.6%, respectively). Furthermore, the KZN province had high rates of overweight in men (23.7%) and women (25.2%) and high rates of obesity (44.0%) [5]. The high prevalence of overweight and obesity observed among South African women is of concern as it can lead to other health conditions, such as arthritis, sleep apnoea, hypertension, DM, CVD, breast cancer, stroke, gall bladder disease, non-alcoholic fatty liver disease, and an increased risk of mortality [22]. The high rates of obesity seen among the South African population could be partly attributed to the nutrition transition, whereby refined diets high in sugar, fat, and salt have replaced traditional diets high in fibre [12,23,24]. As mentioned earlier, there is a paucity of data on the nutritional status of specific rural communities in SA. Thus, it would be beneficial to investigate the nutritional status of these population groups within KZN to establish a baseline nutritional status for possible food-based nutrition interventions.

In most sub-Saharan African countries, including SA, most rural communities consume a limited variety of food and inadequate fruit and vegetables [25]. Poverty is a major contributing factor to inadequate food intake as one in every two South Africans is living in poverty, which equates to approximately 30.4 million South Africans [26]. Furthermore, South Africans from the African ethnic group are particularly affected by poverty (47–53%) [27]. The largest proportion of economically disadvantaged households is found in the KZN province, one of the poorest provinces in SA [26,27,28]. Economically disadvantaged households are at a high risk of malnutrition, as they cannot afford a diverse diet [29,30]. Within KZN, in 2018, 51.9% of economically disadvantaged individuals relied on social grants as a source of income, with the Child Support Grant (CSG) being an important income source [28]. Currently (2021), the CSG is ZAR 460/month (USD 32/month), a ZAR 30 (USD 2) increase from 2020, and is inadequate as a sole source of income to buy food. [31,32]. A basic food basket in SA in 2021 costs approximately ZAR 974.80/month (USD 68.27/month) and may be unaffordable to many economically disadvantaged people [33]. Several strategies have been proposed to address dietary diversity in economically disadvantaged rural households, including biofortification and the promotion of underutilized crops that are nutrient-dense [30].

Under- and over-nutrition co-exist in SA. Children are affected by stunting and over-nutrition, whereas adults, especially females, are obese. The diets consumed, especially by the African rural communities, lack dietary diversity. The studies described earlier aimed to determine the nutritional status of the South African population. However, dietary intake and nutritional status vary across the South African provinces and defined population groups within a province. Nutrition data from specific provinces and defined areas (or population groups) within a province are required to implement targeted nutritional interventions. From the available literature, it seems that detailed studies of the dietary intake of defined population groups living in specific areas of KZN have not been conducted. To address this information gap, the current study aimed to assess the nutritional status of four selected rural communities in KZN where limited studies have been conducted, using selected anthropometric indices and dietary assessment methods.

## 2. Materials and Methods

### 2.1. Study Design

A cross-sectional study design was applied in the current study. Quantitative descriptive data were collected using a survey. Purposive sampling was used to generate a sample of 50 households each from Swayimane, Umbumbulu, and Tugela Ferry and 21 households from Fountain Hill Estate. As alluded to earlier, the double burden of malnutrition is prevalent in most parts of SA, including the KZN province. However, the nutritional status of populations living in certain areas of KZN is still not known. There is no published data on individuals’ nutritional status or dietary patterns residing in the selected research sites. Therefore, it was imperative to determine this.

Furthermore, these study sites were selected as they represented different bioclimatic regions. This was important in formulating a food-based approach that would introduce biofortified and indigenous crops into the study sites, thus emphasizing the importance of agriculture. Anthropometric measurements (height, weight, mid-upper arm circumference (MUAC), and waist circumference) and dietary intake (24 h repeated recall and food frequency) data were collected. A sample of 18 households was used for the pilot study.

### 2.2. Criteria Used for Selection

All members of selected households present on both days of data collection were included in the study. The following individuals were excluded from the study: pilot study participants, individuals who were not at home on either of the two days of data collection, and children who were at day-care, crèche, or on a school feeding program.

### 2.3. Anthropometry

Anthropometric measurements were taken from all household members who met the inclusion criteria, and results were separated into three categories: children (≥1 and ≤5 years), adults, and women of childbearing age (16–35 years). Children who were not at a day-care or crèche on the day of data collection or had not attended a school feeding programme the day before were included in the study. Weight, height, and MUAC data were collected from children ≥1 and ≤5 years, and weight, height, and waist circumference measurements were collected from adult participants.

The weight of the study participants, both children and adults, were measured with a Seca 813 scale (GmbH & Co. KG., Hamburg, Germany). All scales were calibrated using a 1 kg packet of porridge at the start of the day before taking any weight measurements and at the end of the day, after all measurements were taken. The scale was set at zero before and after each participant was weighed. Triplicate weight measurements were recorded with an accuracy of 0.1 kg.

Height (in metres) of children (>2 years old) and adult study participants were measured using the Seca 213 portable stadiometer (GmbH & Co. KG., Hamburg, Germany), which had an accuracy of 0.1 cm. The height and weight of each study participant were measured in triplicate, and mean height and weight were calculated. It took between 5 and 15 min to collect anthropometric data from each participant.

#### 2.3.1. Children

##### Weight

All children were weighed with minimal clothing, including diapers. Children wearing diapers had their diapers changed by their caregivers before the weight measurement was taken. The World Health Organization (WHO) cut-offs were used to classify weight-for-age (WFA) and weight-for-height (WFH) in this study [34]. The WFA and WFL/WFH were calculated using the WHO AnthroPlus software (version 3.1). The WHO classification of nutritional status was used to categorise the degree of underweight and malnutrition in children.

##### Length/Height

The length was measured with a Seca 210 (GmbH & Co. KG., Hamburg, Germany) mobile measuring mat for babies and toddlers (<2 years old). The mobile measuring mat was placed on a level sturdy surface before the measurement was taken. The caregiver was asked to remove the child’s socks, shoes, and hair ornaments before the child was placed on the mobile measuring mat. The caregiver placed the child on the mat with the child’s head held in a straight position. The research assistant ensured that the child’s head was placed against the board. The research assistant then used one hand to hold the child’s legs down and the other hand to move the footboard towards the child’s heels. The research assistant ensured that the child’s knees were not bent during this procedure. The caregiver assisted the research assistant when needed.

When the research assistants measured the standing height, the child stood with their feet slightly apart and their bodies in an upright position. The research assistant ensured that the child was relaxed, with their arms at their side. The back of the child’s head, shoulders, and buttocks were against the board, and knees were straight, with the child looking straight ahead. The research assistants ensured that the head was in the Frankfort Plane before the measurement was taken [35]. The child took in a deep breath, and the headboard was moved down until it rested firmly on the child’s head. For children who could not follow commands, the research assistant pressed the child’s tummy gently and moved the headboard down. The height-for-age (HFA) was determined using the height or length measurements and classified using the WHO classification [36]. The HFA was calculated using the WHO AnthroPlus software (version 3.1). The WHO status for classification of nutritional status was used to classify the degree of stunting in children.

##### Mid-Upper Arm Circumference (MUAC)

The MUAC was taken by research assistants using a Seca 201 fibreglass non-stretch measuring tape (GmbH & Co. KG., Hamburg, Germany). The measurement was taken halfway between the acromion process and the tip of the olecranon process of the left arm. Research assistants ensured that the measuring tape was not too tight or loose when the measurement was taken. All measurements were taken in triplicate to the nearest 0.1 cm and classified using the WHO guidelines to assess the MUAC [37].

#### 2.3.2. Adults

##### Weight and Height

For the weight measurement, the participant was required to stand in the middle of the scale with their feet slightly apart and as still as possible. Similar to the children, all heavy objects in pockets, excess clothing and shoes were removed from the adult study participants before the participant was weighed. Height measurements were taken using the same procedure used to take standing height measurements in children.

##### Body Mass Index (BMI)

Mean weight and height measurements were used to determine BMI for individuals above the age of 18 years. The BMI was calculated using the equation, BMI = weight (kg)/height (m)^2^. The BMI helps determine an individual’s nutritional status and risk for obesity-related conditions [5,38].

##### Waist Circumference

Waist circumference measurements were taken at the umbilicus level for all participants. A Seca 201 fibreglass non-stretch measuring tape (GmbH & Co. KG., Hamburg, Germany) was placed in a horizontal plane over the navel of the participants, who wore a light layer of clothing when the measurements were taken. Waist circumference measurements were taken to the nearest 0.1 cm, in triplicate. A waist circumference greater than 88 cm in females and greater than 102 cm in males indicates risk for comorbidities, including obesity [38,39]. This classification was used to determine the risk for comorbidities in this study. The waist circumference data used in the current report were fewer than the data for BMI because some of the waist circumference measurements were either missing or not taken correctly (n = 4). Incorrectly taken measurements were discarded.

### 2.4. Dietary Intake

#### 2.4.1. 24 h Repeated Recall Method

In this study, a 24 h repeated recall method was used to assess the dietary intake of the target group at a nutrient level. Trained research assistants interviewed the selected household members to obtain information about the types of foods consumed by individuals over the age of one year, over 24 h for two non-consecutive days. This data set was collected randomly on all days of the week, including weekends. There was no specific day allocated for each research assistant to collect data. Data was collected on any day of the week as long as it was two non-consecutive days. The primary caregiver was interviewed in the case of children under four years of age. Children between 4–8 years of age were interviewed together with their primary caregiver. The information obtained from the 24 h recall included portion sizes, preparation methods and ingredients used. Portion sizes were determined using measuring cups, plates of different sizes, cups, mugs, glasses, measuring spoons, dishing spoons, and rulers. The number of 24-repeated recalls obtained from each household varied depending on the number of individuals living in that particular household. The first step of the 24 h recall was to determine the time of day and place that the food was consumed. The participants were then asked to list the foods that they consumed at times documented. After that, participants were asked to describe the preparation methods used to prepare the meals. Participants then provided the research assistants with household measures used to serve the meals to determine portion sizes. Participants indicated to the research assistant whether this was the usual diet followed. Lastly, participants and caregivers were asked if they gave their children any vitamins or mineral supplements, including those collected from clinics or hospitals, so that they could be included in the analysis of dietary intake. The 24 h recall was conducted using standardized methods, similar to previous studies [40,41,42,43,44]. It took the research assistants between 20–40 min to obtain each 24 h recall.

#### 2.4.2. Qualitative Food Frequency Questionnaire

A qualitative FFQ was used to collect data to validate the 24 h recall data in the current study. The FFQ comprised 94 food items subdivided into the following groups: cereals and grains, bread, biscuits and snacks, starchy vegetables, starchy foods prepared with fats, fruit, milk, milk products, vegetables, meat and meat substitutes, fats, and other carbohydrates. Foods included in the FFQ were commonly consumed food items in SA and culturally acceptable food items in KZN. The frequency was indicated next to each food item listed. The frequency options were: never or less than once a month, 1–3 times a month, 2–4 times a week, 5–6 times a week, 7 times a week, 2–3 times a day, 4–5 times a day, or more than 6 times a day. The FFQ that was developed was similar to those used in other studies [45,46]. The trained research assistants recorded the responses of the study participants in the FFQ. The FFQ data were collected from either the head of the household or the person responsible for purchasing the groceries for the household.

### 2.5. Data Analysis

#### 2.5.1. Anthropometry

Data was captured onto password-protected Microsoft Excel spreadsheets and was cross-checked to ensure accuracy of data entry. All data were entered into two password-protected computers. The means and standard deviations for weight, height, MUAC and waist circumference were calculated using Microsoft Excel. The BMI results obtained were coded and interpreted using the BMI classification [34,47], and the WFA, HFA and HFA were interpreted using the WHO classification [34,36].

#### 2.5.2. Dietary Intake Data

The repeated 24 h recall data was captured onto the Food Finder version 3 software programme of the Medical Research Council (MRC), SA, to determine the nutrients consumed over the two non-consecutive days. The foods consumed were recorded each day for each individual, and a report containing an average daily nutrient intake of the two days was generated. Nutrient values for fortified maize and bread were obtained from Food Finder. Data from Food Finder was exported to Microsoft Excel, and the mean daily nutrient intakes and standard deviations were calculated. Of the four Dietary Reference Intakes (DRIs), the Estimated Average Requirement (EAR) and Adequate Intake (AI) were used to assess the nutrient intake. The EAR was selected as the recommended DRI for assessing the nutritional status of population groups, defined by demographic profiles, including age, gender and lifecycle stage [48]. The EAR is the amount of a nutrient estimated to meet the needs of 50% of people in a defined population group.

The percentage of EAR was calculated using the mean intake value for each nutrient compared to the EAR for that value. Thereafter, the prevalence of inadequate intake was determined using the EAR cut-point method. The EAR cut-point method can be used for most nutrients, but not for energy [49,50]. Therefore, the Estimated Energy Requirement (EER) was calculated. Average energy values were calculated using sedentary physical activity levels for each age group. The minimum and maximum EER values for normal BMI were used to calculate the average EER for those above 19 years of age. The AI values were used for nutrients without an EAR. The AI is based on observed or experimentally determined estimates of the average nutrient intake of an apparently healthy population group. It is assumed that if the nutrient intake is above the AI, there is a low risk for inadequate intake [48,51]. The mean calcium and vitamin D intake values were compared with the AI values for calcium and vitamin D, respectively, as there was no EAR value available for them. No assumptions were made about the prevalence of inadequacy for calcium and vitamin D as the mean values were below the AI value. Assumptions can only be made for low risk of inadequate intake when they are above the AI. Nutritional analysis was not conducted on the food items obtained from the food frequency questionnaire. An average score was calculated for foods consumed to give an idea of which food items were consumed the least or most. An ordinal scale was used to give an idea of relative frequencies.

### 2.6. Statistical Analysis

Data were analysed using the Statistical Package for the Social Sciences (SPSS) version 25 (SPSS Inc., Chicago, IL, USA). Descriptive statistics, including the means, standard deviations, and frequencies, were computed where applicable. The Chi-square test was used to analyse for relationships among anthropometric data: BMI, WFH, BMI-for-age, and MUAC, and to determine whether there were significant associations among the categorical variable responses selected. The binomial test was used to test whether a significant proportion of respondents selected one of two possible responses. When conditions were not met, the Fisher’s Exact Test was used. The Chi-square test was also used to analyse for associations between anthropometric data and gender and age. The Fisher’s exact test was used to analyse for correlations between MUAC and gender, MUAC and WFH and BMI and waist circumference. A *p*-value of <0.05 was considered statistically significant.

## 3. Results

### 3.1. Demographic Characteristics

The demographic characteristics of the study participants are presented in Table 1. Of the 466 participants (both adults and children) who participated, 63.7% (n = 297) were female, and 42.9% (n = 200) were from Umbumbulu. Most of the participants were between 19–30 years old (23.6%; n = 110) and 31–50 years old (20.2%; n = 94), and the least number of participants were over 70 years old (3.2%; n = 15). One hundred and sixty-five households participated in the study (Table 1).

### 3.2. Anthropometry

#### 3.2.1. Children

Results obtained are presented in Table 2 (weight-for-age classification), (height-for-age classification), (weight-for-length classification), and (MUAC classification) for children 1–5 years (n = 39). There was a significant relationship between MUAC and WFH scores for children from 1 to 5 years of age (Fisher’s Exact = 11.730, *p* = 0.046). A significant number of children with a MUAC below 11.5 cm (37.5%; n = 3) had a WFH score < −3 SD.

#### 3.2.2. Adults

##### Body Mass Index (BMI)

Table 3 presents the BMI distribution for all adults and the BMI classification by gender. The majority of the study participants were either overweight (23.6%; n = 76) or obese (29.5%; n = 95), with a higher prevalence of overweight and obesity among females than males. The prevalence of underweight was higher in males (12.2%) than females (7.2%). A significant percentage of the adult study participants whose BMI was calculated was found to have a normal BMI (122, 37.9%) or were overweight (76, 23.6%) (χ^2^ (5) = 138.137, *p* < 0.0005). The mean BMI was 28.6 kg/m^2^ (SD ± 13.0).

##### Waist Circumference

According to the binomial test, a significant number (67.0%; n = 213) had waist circumference measurements below 88 cm and 102 cm for females and males, respectively (*p* < 0.05). There was a significant relationship between gender and waist circumference [χ^2^ (1) = 53.327, *p* < 0.0005]. A significant proportion of males (92.9%; n = 105) had a normal waist circumference and were not at risk for obesity-related diseases, while 47.3% of females (n = 97) were (*p* < 0.005). The mean waist circumference was 81.1 cm (SD ± 25.3).

##### Overweight and Obesity

There was a significant relationship between BMI and risk for comorbidities associated with waist circumference [χ^2^(6) = 98.377; *p* < 0.0005]. Underweight and normal BMI was not associated with risk for comorbidities and obesity. At the same time, individuals classified as obese class I, II, and III had a high risk for comorbidities.

#### 3.2.3. Women of Childbearing Age (16–35 years)

**Figure 1:**
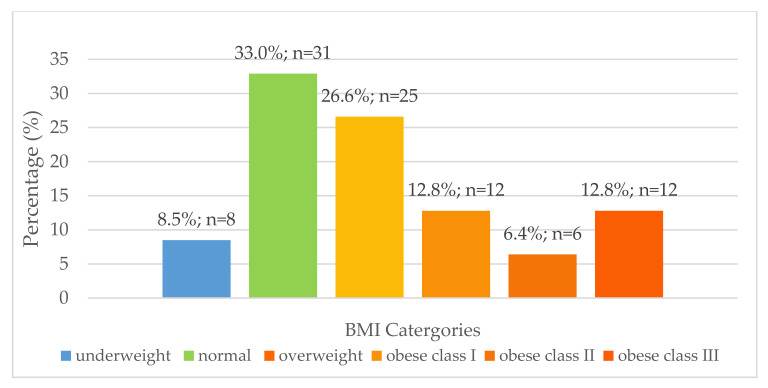
Distribution of body mass index for females aged 16–35 years (n = 94).

Body mass index (Figure 1) and waist circumference were determined for women aged 16–35 years to classify their nutritional status. Although not statistically significant, there was a numerically higher prevalence of over-nutrition than undernutrition at all four research sites for females aged 16–35 years old (Figure 1). Thirty women of childbearing age (32.3%) had a waist circumference of more than 88 cm, and 63 women of childbearing age (67.7%) had a waist circumference of less than 88 cm. There was a significant relationship between BMI and risk associated with waist circumference in women of childbearing age [χ^2^(6) = 23.934; *p* < 0.0005]. Individuals classified as obese class I (n = 6), II (n = 5) and III (n = 8) were at high risk for comorbidities according to waist circumference measurements (*p* < 0.0005).

### 3.3. Dietary Assessment

#### 3.3.1. Dietary Assessment

##### 24 h Repeated Recall

Results from the 24 h repeated recall are presented in Table 4, Table 5, Table 6 and Table 7. The EAR/AI values used to make comparisons are presented as supplementary information (Tables S1 and S2). The average EER value for specific age groups was compared to the mean energy value for that age group to determine the percentage of EER met for each age group (Table 8). Table S3 (Supplementary information) indicates the prevalence of inadequate and adequate nutrient intake for each age and gender group.

The prevalence of inadequate calcium and vitamin D intake could not be determined for all groups as the mean values for these nutrients were below the AI value, except for the 9–13-year-old males. From the results, it can be assumed that males aged 9–13 years consumed adequate amounts of vitamin D.

##### Food Frequency Questionnaire Results

Detailed results of the food items consumed by households and the frequency of consumption are presented as supplementary information (Table S4). Table S5 (Supplementary information) indicates the average frequency score using an ordinal scale. The mean value was determined using the average frequency scores. These scores were used to identify which food items were consumed the most and the least. The ten most commonly consumed food items are presented in Table 9.

## 4. Discussion

This study aimed to assess the nutritional status of communities residing in four rural areas of KZN using selected anthropometric indices and dietary assessment methods. The results indicate that under- and over-nutrition co-exist in children. Compared to other South African studies, the prevalence of stunting in children (30.8%) in the current study was higher than the 2016 SADHS, which found that 27% of children under five years were stunted [6]. The prevalence of stunting in children in the current study was also higher than that reported by earlier national studies. The 2005 NFCS-FB study indicated that one in five children in SA was stunted, and the SANHANES-1 study showed that 26.5% of South African children aged 1–3 years were stunted [5,20]. This study also found a higher prevalence of stunting in children compared to a study conducted in the UMkhanyakude and Zululand districts of KZN, which found that 24% and 26% of children were stunted, respectively [52].

Stunting is a chronic form of undernutrition classified by an HFA < −2 SD and results from a lack of adequate energy intake for a prolonged period [36]. Environmental factors that can contribute to stunting include a lack of hygiene and poor sanitation [53]. There are many negative consequences to stunting that can lead to poor performance in school and poor social and mental development, resulting in a poor quality of life. Furthermore, stunting could lead to obesity in adulthood, which increases the risk for health-related conditions [11,18,54]. The effects of stunting become irreversible after two years of age [54].

This study found that 15.5% (n = 6) of children under five years were overweight. This result was expected as Southern Africa accounts for 12% of overweight children in Africa [2]. The number of overweight children under five years in the current study was higher than other national studies [5,6,20]. The 2005 NFCS-FB found that one in ten South African children were overweight [20], whereas the 2016 SADHS found that 13% of children under five years old were overweight [6]. The percentage of overweight children increased between 2005 and 2016 [5,20]. Furthermore, the 2012 SANHANES-1 study indicated that KZN was one of the provinces with the highest rates of obesity among children [5]. The current study results suggest that childhood overweight and the risk of obesity are of increasing concern in SA, especially in KZN. Childhood obesity has been linked to several chronic diseases in adulthood, such as CVD, hypertension, and DM [18].

It is important to remember that caregivers are responsible for the types of food and portion sizes given to children [55]. Food choice is influenced by many factors such as affordability, seasonality, cultural practices, and personal preferences [29,56,57,58]. Caregivers are more likely to give their children an affordable food item that can positively affect their health [59]. Thus, if caregivers are educated on nutritious foods, they would be able to make more informed choices, leading to improved nutritional status for their children. A good nutritional status can be achieved by having access to nutritious foods and the correct utilization of these foods. Many rural communities cannot afford a variety of foods. However, if healthier, cheaper food types were provided, such as underutilized and biofortified crops and caregivers were educated on the correct processing and preparation methods of these crops, the nutritional status of vulnerable children could be improved. Although the study results indicated a high prevalence of stunting and risk of obesity in children, these results should be interpreted with caution because a small sample was taken from the population of children under five years of age. Further, the Global Acute Malnutrition prevalence was not determined for the small sample size. Another limitation was that anthropometric data were collected only from children present at the study time, as convenience sampling was used to recruit participants from each household.

Unlike with children, overnutrition was prevalent in adults. The prevalence of obesity among females (39.1%) in the current study is similar to the 2016 SADHS, which found that one in five women were severely obese, and the SANHANES-1 study found that 31.8% of females living in rural areas were obese [5,6]. African women have been previously reported to be most at risk of obesity [60,61], similar to the current study. The prevalence of overweight and obesity was higher in females than males in the present study, similar to other SA national studies [5,6]. The current study results reiterate that overweight and obesity are serious public health concerns—they can increase the risk for non-communicable diseases such as CVD, certain cancers, DM, and musculoskeletal disorders [62,63].

Waist circumference measurements and BMI confirmed that females were more at risk for chronic diseases of lifestyle than males. This is a concern as obesity affects women at every stage of the life cycle [64]. It can have economic, biological, and psychosocial implications. A child born to a pregnant, obese mother is at increased risk of chronic diseases, thus negatively affecting the health of future generations [64]. This study indicated that individuals with a higher BMI also had a higher waist circumference, indicating an increased risk for obesity-related diseases. A study by Zhu et al. (2004), conducted on 8712 white men and women, indicated that a combination of high BMI and waist circumference could increase CVD risk [65]. Another study by Gierach et al. (2014) found a direct relationship between waist circumference and BMI. High amounts of abdominal fat were noted in overweight males and females with normal weight [66]. The risk for comorbidities due to a large waist circumference can be independent of BMI [67,68].

The present study indicated that women were more at risk of overweight and obesity than men. Thus, it is important to investigate the nutritional status of women of childbearing age as their nutritional status directly affects foetal development [64,69]. This study found that among the females aged 16–35 years, there was a higher prevalence of overweight and obesity (n = 55; 58.5%) than underweight (n = 8; 8.5%). This was an expected result for rural areas of KZN as the 2012 SANHANES-1 study reported that, on a national level, the prevalence of obesity in women from the rural formal and informal rural populations was 31.8% and 37.6%, respectively [5]. Furthermore, the SANHANES-1 study found that the prevalence of overweight and obesity was high among women (24.8% and 39.2%, respectively), which was similar to findings of the current study (26.6% and 31.9%, respectively). Additionally, the prevalence of overweight and obesity has been found to increase with age in African females [17,65,70,71]. This study assessed the nutritional status using selected anthropometric indices, without including body composition. It would have been also useful to assess body composition using the bioelectrical impedance vector analysis (BIVA) technique; unfortunately, that was not carried out due to resource constraints. The use of BIVA to assess body composition should be explored in future studies.

A possible reason for the increase in obesity could be due to incorrect perceptions. Many women residing in rural areas often do not perceive themselves as overweight or obese and do not consider their weight a problem [70,71,72]. Another reason could be the negative stigma associated with thinness. In some cultures, thinness is associated with being ill, whereas obesity is associated with wealth, happiness, and good health [70,71].

The results of this study indicate that there is a need to address obesity, especially in KZN. It is challenging to address incorrect perceptions of body weight as rural African women have strong cultural beliefs [70]. In most cases, individuals that are most affected by overweight and obesity are women that have less than a primary school education [73]. Thus, nutrition education has the potential to contribute to improving the nutritional status of these communities. Women are usually responsible for preparing meals and for feeding children. If nutrition education is targeted at women, and they are taught how to modify their diets or prepare nutritious underutilized crops, they are more likely to prepare this for themselves and their families, resulting in an improved nutritional status [55].

Before diets can be modified to improve their nutritional composition, it is important to determine the dietary intake of the target population. The results from the 24 h repeated recall suggest that not all age groups met their nutritional requirements. Large quantities of foods containing protein and carbohydrates were consumed, whilst the diet did not have adequate amounts of dietary fibre in most age groups. All age groups met between 62.17–89.26% of the average EER. The maximum energy intake for all age groups was higher than the average EER for all age groups. Although protein intake was high, it was obtained mainly from plant sources rather than animal sources. These results are similar to Kolahdooz et al. (2013), where most of the study participants obtained protein from plant source foods. Animal sources of protein are known to contain appreciable amounts of essential micronutrients, fatty acids, and high-quality protein [74]. The low intake of animal proteins found in the current study could be attributed to these food sources’ limited availability and accessibility, especially in rural areas [75]. Many rural communities do not have access to a variety of foods and rely solely on Spaza shops (informal convenience shops found in rural areas that sell a small range of food items). These shops do not sell a variety of food items; making it worse, the food items are sold at exorbitant prices [76]. Many rural households purchase starchy foods such as maize meal; these foods are cheaper when bought in bulk [77,78]. A review conducted by Schönfeldt and Hall (2012) [74] shows that plant-based, shelf-stable, starchy staples are a predominant part of the diet in disadvantaged communities. In this study, the food frequency confirmed that *phutu* (n = 158), made from maize meal, was one of the most frequently consumed food items.

Starchy foods consumed alone are not nutritionally adequate. However, animal protein sources are unaffordable to most people. Legumes could be considered a good alternative to animal protein sources [79]. It contains protein and fibre, which contributes to satiety, resulting in the consumption of smaller portions of food [80], thus preventing overeating and weight gain. However, to increase the quality of dietary plant protein, the concept of complementary protein is recommended. When a cereal grain food is consumed together with a legume, they complement each other in protein quality. The starchy cereal grains are generally high in the essential amino acid methionine but deficient in the two essential amino acids, lysine and tryptophan. In contrast, legumes are high in the two amino acids [81]. Thus, composite foods comprised of cereal grains and legumes would be an affordable source of quality protein.

Additionally, more than 50% of the sample of children and adults of the current study had inadequate intake of the following nutrients: magnesium, phosphorus, zinc, riboflavin, niacin, folate, vitamin B12, vitamin A, vitamin C, vitamin E, and vitamin K, for most of the age groups. These results were similar to results from the 1999 NFCS, which indicated that South African children, especially rural children, had a low intake of the following micronutrients: vitamin A, calcium, iron, zinc, folate, vitamin B6, niacin, riboflavin, vitamin C, and vitamin E [8]. Possible reasons for the inadequate nutrient intake could be due to the seasonal availability of foods or foods not consumed at the time of data collection. The current study results should be interpreted with caution as 24 h recalls were only collected for two days. Dietary data is very subjective, and there is room for inaccurate reporting and analysis [82].

Micronutrient deficiency is defined as an inadequate status of one or more micronutrients and is just as serious as overnutrition. Although micronutrient deficiency is a problem, it is not routinely treated in developing countries. It can result in several health conditions, including growth retardation and delayed development [14]. Thus, individuals need to consume good quality, micronutrient-dense foods, but this is not always possible due to the high cost of these foods. The most common micronutrient deficiencies observed in developing countries are iron, iodine, zinc, and vitamin A [83]. Amongst the micronutrient deficiencies, iron and vitamin A are of concern in SA. Many South African national studies have shown this to be true, especially in vulnerable groups such as women and children [5,8,9,14,18].

The current study results indicate that 50% of the females aged 14 to >70 years had an inadequate iron intake. The low intake could be due to the high cost of iron-rich foods. As mentioned earlier, many rural communities rely predominantly on starch-based diets, which contain limited amounts of fruits, vegetables and animal protein [5]. A study conducted in 2016 on 651 healthy South African adults indicated that the prevalence of anaemia was 12.6%, which was lower than the findings of the 2012 SANHANES-1 study [84]. Further, both studies found the prevalence of anaemia higher among females than males [5,84]. This study showed that dietary intake of iron was low, and females were at higher risk of becoming iron deficient or further worsening their already poor iron status. This is a major concern, especially for pregnant women, as iron requirements are increased during pregnancy. If a mother has a poor nutritional status, she is at increased risk of giving birth to a malnourished child [85]. Furthermore, if a mother has iron-deficiency anaemia during pregnancy, it can result in fetal and maternal mortality, morbidity, pre-eclampsia, bleeding, and infection [69].

In this study, vitamin A intake was inadequate in all age groups due to poor intake of animal-based foods, fruits, and vegetables. Furthermore, no participants had reported that they were taking any vitamin or mineral supplements at the time of data collection. Thus, the analysis of vitamin A did not include any vitamin A supplements. The low intake of dietary vitamin A observed in this study suggests that the communities were at high risk of vitamin A efficiency (VAD) and/or were already experiencing it. The low intake of dietary vitamin A found in the current study is in line with national data, which indicated that the vitamin A status of South African children had worsened between 1994 and 2005 [8,9,20].

Further, the 2005 NFCS-FB study indicated that one in ten women were vitamin A deficient in KZN [20]. The 2012 SANHANES-1 study reported high rates of VAD throughout SA; however, it noted an improvement in the status of women residing in KZN [5]. The South African government has employed several interventions to combat VAD, including food fortification, vitamin A supplementation and dietary diversity [74,75,76]. The current study indicates that although these interventions are in place, vitamin A intake remains inadequate. As mentioned previously, there are some limitations with the use of the 24 h recall method. It relies on recent memory and an individual’s ability to recall details about all food eaten over a 24 h period. It requires well-trained interviewers, and a single 24 h recall cannot be used to estimate usual intake [77]. To overcome these limitations, all research assistants were trained on how to take the 24 h recall. The 24 h recall was collected over two days, and an FFQ interview was conducted to validate the 24 h recall.

The food frequency results indicated that onion, *phutu*, brown bread, tomato, rice, apple, eggs, and chicken were the most commonly consumed food items. This was similar to studies conducted in SA, which documented that mealie meal, white sugar, tea, brown bread, non-dairy creamer, brick margarine, chicken meat, full cream milk and dark green leafy vegetables were frequently consumed [8,78]. Similarly, another study conducted in KZN by Faber et al. (2013) found that sugar, maize meal porridge, bread, rice, cordial squash, hard margarine, tea, and legumes were the most commonly consumed food items. Additionally, a study conducted by Faber et al. (2015) found that 50.5% of participants from rural KZN consumed both bread and maize meal [79]. From this, it is evident that in SA, more specifically in rural areas of KZN, most diets comprise of starchy foods, either a maize-based dish, bread, or rice. This could lead to an excessive intake of carbohydrates and energy, thus contributing to a high prevalence of obesity [64].

Consumption of high amounts of refined carbohydrates has been associated with an increased risk of coronary heart disease, insulin resistance, obesity, DM, hypertension, and stroke [72,80,81]. Study participants who were overweight and obese were at increased risk for CVD, certain cancers, DM, and hypertension. A possible reason for this high prevalence could be due to poor food choices, a sedentary lifestyle, or a lack of physical activity [64,82,83]. Increasing physical activity and energy expenditure could reduce the rates of obesity [83]. However, this study did not investigate physical activity. Most study participants consumed processed foods or purchased foods prepared with large amounts of fat and sugar, resulting in high energy intake, which could be a contributing factor to the high rates of overweight and obesity observed. Sweets were one of the most commonly consumed unhealthy food items in this study. If more fruit were consumed in place of high-energy snacks, and if preparation methods such as steaming, baking, and boiling, rather than frying, were incorporated into daily cooking, this could reduce the prevalence of obesity in the long term [82,86]. Rural communities could achieve this if they saved the money usually spent on unhealthy snacks to buy fruit in season and cheaper. A limitation of using an FFQ is that it does not always provide the most accurate information as participants can under or over-report the consumption frequency of food items [87]. Thus, the FFQ was used together with the 24 h recall to validate the results.

## 5. Conclusions

The study’s objective was to assess, by a survey, the nutritional status using selected anthropometric indices and dietary intake methods of four rural communities in KZN, SA. No published nutritional studies have been conducted at the sites selected for this study, and there was no baseline nutritional data available for the target population. Therefore, it was necessary to determine these communities’ nutritional status and dietary patterns so that appropriate food-based approaches to address malnutrition could be then investigated.

The current study results indicate that under- and over-nutrition co-exist in the African rural communities of KZN studied. Stunting and the risk of obesity were prevalent among children under five years of age, whilst obesity affected adults, especially females aged 16–35. Future studies should be conducted over a longer period to collect anthropometric data from children at home, in schools and at crèches to assess more children in the target areas. The prevalence of obesity could be reduced by educating the affected population groups on optimum food preparation methods and processing and emphasizing healthier, cheaper food types and portion size control. As part of community initiatives or community resource programmes, a potentially sustainable strategy could be introducing biofortified and underutilized crops, which would be accessible and affordable to the vulnerable communities. The target communities would be taught how to utilize these crops to improve the nutritional composition of their diets. However, the acceptance of alternative food types would need to be investigated.

Dietary intake plays a vital role in determining nutritional status. The dietary intake patterns indicated frequent consumption of food items high in carbohydrates and low in fibre and micronutrients such as vitamin A. The findings suggest that the nutrition transition has influenced the nutritional status of the population groups living in rural KZN. From these results, it is clear that there is a need to develop a food-based approach to address the double burden of malnutrition affecting these communities. The use of agriculture to improve the availability of and access to diverse, affordable, and nutrient-dense foods should be explored. In this regard, the inclusion of several nutritious underutilized crops in such agricultural interventions should be encouraged. Similar studies should be conducted in the future, but using larger samples of the selected populations to draw firmer conclusions. In addition, future studies should explore improving current diets with PVA-biofortified maize and OFSP and underutilized protein-rich crops such as Bambara groundnut. Furthermore, there is a need to investigate the nutritional composition of these crops so that these crops can be incorporated into the diets of vulnerable population groups to improve their nutritional status.

## Figures and Tables

**Table 1:** Demographic characteristics of study participants.

Characteristics	n	%
Gender *		
Male	169	36.3
Female	297	63.7
Participant distribution by study site *		
Swayimane	139	29.8
Umbumbulu	200	42.9
Tugela Ferry	114	24.5
Fountain Hill Estate **	13	2.8
Age (years) *
1–3	16	3.4
4–8	59	12.7
9–13	51	10.9
14–18	50	10.7
19–30	110	23.6
31–50	94	20.2
51–70	71	15.2
70+	15	3.2
Household distribution ***
Swayimane	50	30.3
Umbumbulu	53	32.1
Tugela Ferry	49	29.7
Fountain Hill Estate	13	7.9

* Percentage of sample (n = 466); The distribution of participants is different in each research site as each household had a different number of participants; ** All participants from Fountain Hill Estate were adult farmworkers; *** Percentage of sample (n = 165).

**Table 2:** Weight-for-age, height-for-age, weight-for-height, and MUAC classification in children 1–5 years (n = 39).

Weight-for-Age Classification		Height-for-Age Classification		Weight-for-Height Classification		Mid-Upper Arm Circumference	
Classification	n (%)	Classification	n (%)	Classification	n (%)	Classification	n (%)
Severely underweight	2 (5.1)	Severely stunted	8 (20.5)	Severely wasted	3 (7.7)	Severe acute malnutrition (SAM)	8 (20.5)
Moderately underweight	5 (12.8)	Moderately stunted	4 (10.3)	Moderately wasted	1 (2.6)	Moderate acute malnutrition (MAM)	2 (5.1)
Normal weight	25 (64.1)	Normal height	26 (66.7)	Not wasted	29 (74.4)	Not acutely malnourished (NAM)	29 (74.4)
Overweight	7 (17.9)	Tall *	1 (2.6)	Overweight	6 (15.4)		

* Children under five years with a height-for-age (HFA) greater than the +3 SD were classified as tall.

**Table 3:** Body mass index classification for adults ≥ 18 years (n = 322).

	BMI Classification (n = 322)					
Gender	Underweight (<18.5 kg/m^2^)	Normal	Overweight	Obese Class I	Obese Class II	Obese Class III
		(18.5–24.9 kg/m^2^)	(25.0–29.9 kg/m^2^)	(30.0–34.9 kg/m^2^)	(35.0–39.9 kg/m^2^)	(≥40.0 kg/m^2^)
Male (n = 115)	14 ^a^ (12.2%) ^b^	65 (56.5%)	22 (19.1%)	9 (7.8%)	3 (2.6%)	2 (1.7%)
Female (n = 207)	15 ^c^ (7.2%) ^d^	57 (27.5%)	54 (26.1%)	33 (15.9%)	20 (9.7%)	28 (13.5%)
Total (n = 322)	29 (9.0%)	122 (37.9%)	76 (23.6%)	42 (13.0%)	23 (7.1%)	30 (9.3%)

^a^ Number of males; ^b^ Percentage of males; ^c^ Number of females; ^d^ Percentage of females; BMI, Body mass index.

**Table 4:** Mean nutrient (SD) intake from two 24 h repeated recalls for individuals aged 1–70 years.

**Nutrients**	**Age (Years), Gender**					
	**1–3**	**4–8**	**9–13, Female**	**9–13, Male**	**14–18, Female**	**14–18, Male**
	**(n = 16)**	**(n = 59)**	**(n = 37)**	**(n = 14)**	**(n = 32)**	**(n = 18)**
Total protein (g)	28.2 ± 30.0	35.2 ± 14.0	45.7 ± 20.2	47.5 ± 21.0	43.6 ± 17.0	43.1 ± 21.2
Carbohydrates (g)	105.9 ± 46.4	166.6 ± 51.3	183.6 ± 52.6	201 ± 57.7	214.2 ± 63.3	198.9 ± 64.3
Dietary fibre (g)	8.6 ± 4.1	11.7 ± 7.0	30.7 ± 52.2	17.9 ± 7.8	17.4 ± 9.4	15.2 ± 9.5
Calcium (mg)	165.3 ± 89.0	297 ± 209.2	279.5 ± 240.8	334.2 ± 255.8	298.6 ± 205.0	325.9 ± 344.1
Magnesium (mg)	115.4 ± 58.6	157.6 ± 58.3	201.5 ± 99.8	225.4 ± 50.9	204 ± 58.5	199 ± 76.0
Phosphorus (mg)	416 ± 287.8	578.1 ± 239.4	722.5 ± 338.2	764.6 ± 308.1	703.3 ± 255.1	684.2 ± 276.2
Iron (mg)	3.8 ± 2.6	5.7 ± 3.3	14.3 ± 32.3	7.6 ± 2.8	7.8 ± 3.6	6.3 ± 3.1
Zinc (mg)	4 ± 4.4	4.2 ± 1.6	14.3 ± 36.0	5.5 ± 2.0	5.6 ± 2.1	6.1 ± 2.7
Thiamine (mg)	0.5 ± 0.3	0.7 ± 0.3	0.8 ± 0.3	4.4 ± 13.4	0.9 ± 0.4	0.7 ± 0.3
Riboflavin (mg)	0.4 ± 0.2	0.6 ± 0.4	0.8 ± 1.0	0.6 ± 0.4	0.7 ± 0.5	0.6 ± 0.4
Niacin (mg)	6.5 ± 6.8	9.2 ± 5.0	12.3 ± 7.1	11.2 ± 8.3	11.8 ± 6.7	9.4 ± 6.6
Vitamin B6 (mg)	0.6 ± 0.4	0.9 ± 0.5	1.3 ± 0.7	1.4 ± 1.1	1.2 ± 0.49	1 ± 0.5
Folate (µg)	88.8 ± 54.6	142.3 ± 92.8	193.8 ± 100.8	156.2 ± 85.3	202.7 ± 104.5	159.3 ± 91.9
Vitamin B12 (µg)	2.3 ± 3.1	3.3 ± 4.6	9 ± 26.2	23.1± 64.2	3.6 ± 5.6	2.1 ± 2.9
Vitamin C (mg)	32.3 ± 36.1	57.5 ± 154.1	171.8 ± 365.1	51.1 ± 96.0	104.6 ± 258.5	57.4 ± 156.7
Vitamin A (µg)	198 ± 205.1	183.1 ± 111.6	176.1 ± 129.7	250.5 ± 187.7	236 ± 165.3	225.4 ± 153.0
Vitamin D (µg)	2.7 ± 3.0	3.1 ± 3.5	4 ± 4.4	6.9 ± 7.4	4.1 ± 5.3	2.5 ± 2.7
Vitamin E (mg)	2.8 ± 2.9	5.9 ± 4.3	5.8 ± 3.5	4.9 ± 2.9	7.4 ± 5.7	6.2 ± 4.5
Vitamin K (µg)	16.2 ± 22.3	76.1 ± 118.6	58.1 ± 103.2	168.4 ± 212.2	143.1 ± 273.1	103.9 ± 218.2
**Nutrients**	**Age (Years), Gender**					
	**19–30, Female**	**19–30, Male**	**31–50, Female**	**31–50, Male**	**51–70, Female**	**51–70, Male**
	**(n = 67)**	**(n = 43)**	**(n = 62)**	**(n = 32)**	**(n = 51)**	**(n = 20)**
Total protein (g)	49.8 ± 17.9	51 ± 22.4	56.4 ± 26.0	59.7 ± 33.1	40.8 ± 20.4	52.7 ± 21.3
Carbohydrates (g)	205.8 ± 62.3	222 ± 49.3	225.3 ± 74.8	224.1 ± 98.5	209.3 ± 53.2	244.8 ± 103.3
Dietary fibre (g)	15.3 ± 5.9	18.9 ± 10.5	25.9 ± 63.2	19.3 ± 15.1	18.4 ± 8.4	19.7 ± 9.9
Calcium (mg)	312.7± 240.5	283.8 ± 192.2	348 ± 256.6	307.9 ± 252.8	347.3 ± 380.6	264.8 ± 150.5
Magnesium (mg)	209.5 ± 58.9	220.2 ± 56.4	235.4 ± 84.8	248.2 ± 102.1	232.6 ± 54.9	241.3 ± 93.0
Phosphorus (mg)	736.5 ± 223.8	758.3 ± 246.3	828.4 ± 312.6	865 ± 399.1	699.1 ± 255.7	793.4 ± 295.0
Iron (mg)	6.8 ± 2.3	6.8 ± 2.5	7.9 ± 3.4	14 ± 33.6	6 ± 2.1	7.1± 2.5
Zinc (mg)	6.7 ± 3.0	6.9 ± 3.0	7.3 ± 3.1	7.7 ± 4.5	5.9 ± 3.6	6.6 ± 2.5
Thiamine (mg)	0.9 ± 0.8	0.9 ± 0.3	0.9 ± 0.3	0.9 ± 0.3	0.8 ± 0.2	0.9 ± 0.4
Riboflavin (mg)	0.8 ± 0.7	0.6 ± 0.3	0.8 ± 0.5	0.8 ± 1.1	0.6 ± 0.4	0.8 ± 0.9
Niacin (mg)	14.8 ± 22.6	11.9 ± 6.3	13.4 ± 8.1	14.6 ± 8.9	8.2 ± 5.2	12.1 ± 5.9
Vitamin B6 (mg)	1 ± 0.5	0.9 ± 0.5	1 ± 0.5	1 ± 0.6	0.7 ± 0.3	0.9 ± 0.4
Folate (µg)	172.5 ± 80.1	173.1 ± 66.0	223.8 ± 141.5	180.9 ± 101.0	170.9 ± 68.2	204.2 ± 115.6
Vitamin B12 (µg)	6.5 ± 17.2	3.2 ± 5.9	4.2 ± 8.2	7.8 ± 24.8	1.7 ± 3.8	7.1 ± 23.7
Vitamin C (mg)	44.3 ± 48.9	149.9 ± 415.1	72.6 ± 143.6	35.8 ± 34.0	70.1 ± 188.2	33.8 ± 24.8
Vitamin A (µg)	231.2 ± 184.2	238.3 ± 134.0	287.3 ± 130.3	235.4 ± 207.3	293 ± 183.5	215.7 ± 141.5
Vitamin D (µg)	3.1 ± 4.0	3.5 ± 4.2	3.6 ± 4.3	4 ± 4.4	1.6 ± 2.3	2.4 ± 2.4
Vitamin E (mg)	5.8 ± 4.3	6.5 ± 5.7	7.2 ± 4.7	7.2 ± 4.0	6 ± 4.9	7.3 ± 7.7
Vitamin K (µg)	118.2 ± 234.0	57.2 ± 121.0	104.1 ± 176.1	166.1 ± 392.1	190.2 ± 265.6	76.6 ± 104.6

**Table 5:** Percentage of EAR met from the two 24 h repeated recalls for individuals aged 1–70 years.

**Nutrients**	**Age (Years), Gender**											
	**1–3**		**4–8**		**9–13, Female**		**9–13, Male**		**14–18, Female**		**14–18, Male**	
	**(n = 16)**		**(n = 59)**		**(n = 37)**		**(n = 14)**		**(n = 32)**		**(n = 18)**	
	**% EAR**	***p*** **Value**	**% EAR**	***p*** **Value**	**% EAR**	***p*** **Value**	**% EAR**	***p*** **Value**	**% EAR**	***p*** **Value**	**% EAR**	***p*** **Value**
Total protein (g)	256	**0.036**	235	**<0.0005**	163	**<0.0005**	176	**0.003**	156	**<0.0005**	98	0.861
Carbohydrates (g)	106	0.618	167	**<0.0005**	184	**<0.0005**	201	**<0.0005**	214	**<0.0005**	199	**<0.0005**
Dietary fibre (g)	45	**<0.0005**	47	**<0.0005**	118	0.589	58	**<0.0005**	67	**<0.0005**	-	**<0.0005**
Calcium (mg)		**<0.0005**		**<0.0005**	-	**<0.0005**	-	**<0.0005**	-	**<0.0005**	-	**<0.0005**
Magnesium (mg)	178	**0.004**	143	**<0.0005**	101	0.926	113	0.084	68	**<0.0005**	59	**<0.0005**
Phosphorus (mg)	110	0.624	143	**<0.0005**	65	**<0.0005**	73	**0.004**	67	**<0.0005**	65	**<0.0005**
Iron (mg)	127	0.216	139	**<0.0005**	251	0.113	129	**0.048**	99	0.872	82	0.071
Zinc (mg)	160	0.199	105	0.272	204	0.224	79	**0.015**	77	**<0.0005**	72	**0.002**
Thiamine (mg)	20	0.400	140	**<0.0005**	114	0.100	629	0.325	100	0.651	70	**0.002**
Riboflavin (mg)	100	0.759	120	0.060	100	0.803	75	0.104	78	0.055	55	**<0.0005**
Niacin (mg)	130	0.404	153	**<0.0005**	137	**0.008**	124	0.329	107	0.527	78	0.111
Vitamin B6 (mg)	150	0.123	180	**<0.0005**	163	**<0.0005**	175	0.100	120	**0.037**	91	0.393
Folate (µg)	74	**0.037**	89	0.149	78	**0.002**	63	**0.001**	61	**<0.0005**	48	**<0.0005**
Vitamin B12 (µg)	329	**0.054**	330	**<0.0005**	600	0.089	1540	0.229	240	**0.041**	105	0.878
Vitamin C (mg)	249	**0.049**	261	0.082	441	0.033	131	0.645	187	0.296	91	0.881
Vitamin A (µg)	94	0.818	67	**<0.0005**	42	**<0.0005**	56	**0.002**	49	**<0.0005**	36	**<0.0005**
Vitamin D (µg)	-	**0.008**	-	**<0.0005**	-	0.161	-	0.363	-	0.337	-	**0.001**
Vitamin E (mg)	56	**0.009**	98	0.856	64	**<0.0005**	54	**<0.0005**	62	**<0.0005**	52	**<0.0005**
Vitamin K (µg)	54	**0.026**	138	0.177	97	0.912	281	0.078	191	0.168	139	0.581
**Nutrients**	**Age (Years), Gender**											
	**19–30, Female**		**19–30, Male**		**31–50, Female**		**31–50, Male**		**51–70, Female**		**51–70, Male**	
	**(n = 67)**		**(n = 43)**		**(n = 62)**		**(n = 32)**		**(n = 51)**		**(n = 20)**	
	**% EAR**	***p*** **Value**	**% EAR**	***p*** **Value**	**% EAR**	***P*** **Value**	**% EAR**	***p*** **Value**	**% EAR**	***p*** **Value**	**% EAR**	***p*** **Value**
Total protein (g)	131	**<0.0005**	111	0.154	148	**<0.0005**	130	**0.026**	107	0.325	115	0.174
Carbohydrates (g)	206	**<0.0005**	222	**<0.0005**	225	**<0.0005**	224	**<0.0005**	209	**<0.0005**	245	**<0.0005**
Dietary fibre (g)	61	**<0.0005**	50	**<0.0005**	104	0.912	51	**<0.0005**	74	**<0.0005**	52	**<0.0005**
Calcium (mg)	-	**<0.0005**	-	**<0.0005**	-	**<0.0005**	-	**<0.0005**	-	**<0.0005**	-	**<0.0005**
Magnesium (mg)	82	**<0.0005**	67	**<0.0005**	89	**0.008**	71	**<0.0005**	93	**0.0005**	73	**<0.0005**
Phosphorus (mg)	289	**<0.0005**	131	**<0.0005**	143	**<0.0005**	247	**<0.0005**	121	**0.002**	137	**0.004**
Iron (mg)	84	**<0.0005**	113	**0.048**	98	0.648	2333	0.186	74	**<0.0005**	118	0.061
Zinc (mg)	99	0.711	73	**<0.0005**	107	0.193	82	**0.044**	87	0.070	70	**<0.0005**
Thiamine (mg)	100	0.957	90	**0.006**	100	0.818	90	0.324	89	**<0.0005**	90	0.271
Riboflavin (mg)	89	0.100	55	**<0.0005**	89	0.225	73	0.209	67	**<0.0005**	73	0.177
Niacin (mg)	135	0.174	99	0.899	122	**0.026**	122	0.104	75	**<0.0005**	73	0.949
Vitamin B6 (mg)	91	0.103	82	**0.041**	91	0.300	91	0.532	64	**<0.0005**	82	**0.021**
Folate (µg)	54	**<0.0005**	54	**<0.0005**	70	**<0.0005**	57	**<0.0005**	53	**<0.0005**	64	**<0.0005**
Vitamin B12 (µg)	325	**0.036**	160	0.206	210	**0.036**	390	0.192	85	0.541	355	0.346
Vitamin C (mg)	74	**0.011**	200	0.243	121	0.491	48	**<0.0005**	117	0.702	45	**<0.0005**
Vitamin A (µg)	46	**<0.0005**	38	**<0.0005**	57	**<0.0005**	38	**<0.0005**	59	**<0.0005**	35	**<0.0005**
Vitamin D (µg)	-	**<0.0005**	-	**0.022**	-	**0.013**	-	0.214	-	**<0.0005**	-	**<0.0005**
Vitamin E (mg)	48	**<0.0005**	54	**<0.0005**	60	**<0.0005**	60	**<0.0005**	60	**<0.0005**	61	0.013
Vitamin K (µg)	131	0.327	48	**0.001**	116	0.531	138	0.511	211	**0.010**	64	0.079

EAR: Estimated average requirement. The mean values in Table 4 have been compared with the EAR/AI for each nutrient for each age group. *p* values in bold font indicate that the mean nutrient value in Table 4 is significantly different from the respective EAR/AI (Tables S1 and S2).

**Table 6:** The prevalence of inadequate nutrient intake for each age and gender group.

**Nutrients**	**Age (Years), Gender**					
	**1–3**	**4–8**	**9–13, Female**	**9–13, Male**	**14–18, Female**	**14–18, Male**
	**(n = 16)**	**(n = 59)**	**(n = 37)**	**(n = 14)**	**(n = 32)**	**(n = 18)**
Total protein (g)	31.3%	3.4%	29.7%	14.3%	21.9%	55.6%
Carbohydrates (g)	50.0%	5.1%	2.7%	0%	3.1%	5.6%
Dietary fibre (g)	100.0%	93.2%	75.7%	92.9%	81.3%	94.4%
Calcium (mg)			-	-	-	-
Magnesium (mg)	6.3%	16.9%	59.5%	28.6%	93.8%	94.4%
Phosphorus (mg)	31.3%	25.4%	83.8%	78.6%	90.6%	88.9%
Iron (mg)	50.0%	33.9%	35.1%	28.6%	53.1%	66.7%
Zinc (mg)	43.8%	54.2%	62.2%	64.3%	81.3%	94.4%
Thiamine (mg)	50.0%	30.5%	48.6%	42.9%	65.6%	83.3%
Riboflavin (mg)	68.8%	52.5%	73%	71.4%	71.9%	83.3%
Niacin (mg)	68.8%	27.1%	37.8%	57.1%	59.4%	77.8%
Vitamin B6 (mg)	43.8%	20.3%	18.9%	21.4%	37.5%	61.1%
Folate (µg)	62.5%	66.1%	81.1%	92.9%	87.5%	94.4%
Vitamin B12 (µg)	50.0%	91.5%	48.6%	50%	46.9%	66.7%
Vitamin C (mg)	37.5%	52.5%	54.1%	78.6%	68.8%	94.4%
Vitamin A (µg)	68.8%	78%	91.9%	92.9%	87.5%	100%
Vitamin D (µg)	-	-	-	-	-	-
Vitamin E (mg)	81.3%	59.3%	78.4%	85.7%	81.3%	88.9%
Vitamin K (µg)	81.3%	67.8%	73%	57.1%	78.1%	77.8%
**Nutrients**	**Age (Years), Gender**					
	**19–30, Female**	**19–30, Male**	**31–50, Female**	**31–50, Male**	**51–70, Female**	**51–70, Male**
	**(n = 67)**	**(n = 43)**	**(n = 62)**	**(n = 32)**	**(n = 51)**	**(n = 20)**
Total protein (g)	31.3%	46.5%	27.4%	34.4%	58.8%	50%
Carbohydrates (g)	3%	0%	1.6%	6.3%	2%	5%
Dietary fibre (g)	92.5%	93%	80.6%	90.6%	84.3%	95%
Calcium (mg)	-	-	-	-	-	-
Magnesium (mg)	74.6%	97.7%	67.7%	87.5%	66.7%	80%
Phosphorus (mg)	26.9%	23.3%	17.7%	100%	37.3%	30%
Iron (mg)	64.2%	37.2%	62.9%	21.9%	82.4%	55%
Zinc (mg)	61.2%	79.1%	46.8%	78.1%	74.5%	80%
Thiamine (mg)	70.1%	65.1%	48.4%	71.9%	70.6%	65%
Riboflavin (mg)	71.6%	86%	62.9%	78.1%	86.3%	90%
Niacin (mg)	47.8%	62.8%	46.8%	50%	82.4%	50%
Vitamin B6 (mg)	52.2%	69.8%	62.9%	68.8%	84.3%	70%
Folate (µg)	95.5%	97.7%	82.3%	90.6%	98%	90%
Vitamin B12 (µg)	53.7%	62.8%	61.3%	65.6%	76.5%	60%
Vitamin C (mg)	77.6%	86%	75.8%	84.4%	74.5%	95%
Vitamin A (µg)	89.6%	100%	88.7%	90.6%	84.3%	100%
Vitamin D (µg)	-	-	-	-	-	-
Vitamin E (mg)	89.6%	86%	88.7%	87.5%	88.2%	80%
Vitamin K (µg)	76.1%	93%	75.8%	81.3%	60.8%	80%

**Table 7:** Comparison of mean nutrient intake with the EAR/AI from two 24 h repeated recalls for female adults above 70 years (n = 10) and male adults above 70 years (n = 5).

Nutrient	Females						Males					
	Mean Nutrient Intake (SD)	EAR ^a–d^	AI ^e^	Percentage of EAR	Prevalence of Inadequacy (%)	*p* Value *	Mean Nutrient Intake (SD)	EAR ^a–d^	AI ^e^	Percentage of EAR	Prevalence of Inadequacy (%)	*p* Value *
Total protein (g)	40.3 (20.1)	38	-	106	40	0.724	45.5 (29.0)	46	-	99	60	0.971
Carbohydrates (g)	167.4 (92.1)	100	-	167	20	**0.046**	190.1 (39.8)	100	-	190	0	**0.007**
Dietary fibre (g)	15.5 (2.9)	25	-	62	100	**<0.0005**	11 (4.6)	38	-	29	100	**<0.0005**
Calcium (mg)	175.5 (90.1)	-	1000	-	-	**<0.0005**	393.8 (378.9)	-	1000	-	-	**0.023**
Magnesium (mg)	233.6 (58.0)	255	-	92	70	0.274	240 (81.7)	330	-	73	80	0.070
Phosphorus (mg)	594.6 (191.2)	580	-	103	50	0.815	849 (505.1)	580	-	257	20	0.300
Iron (mg)	5.8 (1.4)	8.1	-	72	90	**0.001**	7.3 (4.2)	6	-	122	60	0.524
Zinc (mg)	5.1 (1.9)	6.8	-	75	90	**0.017**	7.6 (4.3)	9.4	-	81	80	0.390
Thiamine (mg)	0.8 (0.2)	0.9	-	89	60	0.109	0.8 (0.2)	1.0	-	80	80	0.119
Riboflavin (mg)	0.4 (0.2)	0.9	-	44	100	**<0.0005**	1.8 (3.0)	1.1	-	164	80	0.616
Niacin (mg)	8.1 (6.4)	11	-	74	80	0.179	14.2 (9.3)	12	-	118	40	0.618
Vitamin B6 (mg)	0.6 (0.2)	1.1	-	55	100	**<0.0005**	1 (0.8)	1.1	-	91	80	0.730
Folate (µg)	138.2 (64.3)	320	-	43	100	**<0.0005**	155.8 (149.8)	320	-	49	80	0.070
Vitamin B12 (µg)	0.6 (0.5)	2.0	-	30	100	**<0.0005**	35.2 (70.7)	2.0	-	1760	60	0.353
Vitamin C (mg)	72.1 (127.6)	60	-	120	80	0.771	30.4 (20.5)	75	-	41	100	**0.008**
Vitamin A (µg)	297.3 (177.4)	500	-	60	80	**0.006**	159.6 (141.5)	625	-	26	100	**0.002**
Vitamin D (µg)	0.9 (0.9)	-	15	-	-	**<0.0005**	2.3 (3.2)	-	15	-	-	**0.001**
Vitamin E (mg)	7.4 (4.6)	12	-	62	90	**0.012**	4.7 (2.0)	12	-	39	100	**0.001**
Vitamin K (µg)	473.9 (366.8)	90	-	527	20	**0.009**	269.3 (384.7)	120	-	308	60	0.435

^a^ Institute of Medicine 2003; ^b^ Institute of Medicine 2001b; ^c^ Institute of Medicine 2000; ^d^ Institute of Medicine 1998; ^e^ Institute of Medicine 1997; SD, standard deviation; EAR, estimated average requirement; AI, adequate intake; * *p* values given in bold font indicate that the mean nutrient intake is significantly different from the EAR/AI.

**Table 8:** Comparison of mean energy intake with the EER ^a^ from two 24 h repeated recalls for individuals aged ≥1–≥18 years.

Age and Gender	n	Energy Range (kJ ^b^)	Mean Energy (kJ) (SD ^c^)	Energy Range (kCal ^d^)	Mean Energy (kCal/day)	Average EER ^e^ Requirement (kCal/day)	Percentage of EER Met
1–3 years	16	265 ^f^–11,148 ^g^	3275.90 (2477.65)	63.40–2666.99	783.71	1016.70	77.10
4–8 years	59	669–8965	4735.26 (1591.55)	160.05–2144.74	1132.84	1269.10	89.26
9–13 years, female	37	3088–9688	5529.38 (1508.67)	738.76–2317.70	1322.82	1519.80	87.03
9–13 years, male	14	3892–10,427	5712.79 (1666.23)	931.10–2494.50	1366.70	1686.00	81.06
14–18 years, female	32	690–11,497	5925.85 (2294.11)	165.07–2750.48	1417.67	1689.60	83.91
14–18 years, male	18	2800–8272	5850.28 (1728.84)	669.86–1978.95	1399.59	2251.40	62.17
19–30 years female	67	3213–11,374	6183.39 (1683.39)	768.66–2721.05	1479.28	1974.18	74.93
19–30 years male	43	3445–10,660	6857.67 (1724.57)	824.16–2550.24	1640.59	2301.90	71.27
31–50 years, female	62	2985–12,651	6830.11(2113.41)	714.11–3026.56	1634.00	1974.18	82.77
31–50 years, male	32	2910–12,103	6983.91 (2261.46)	696.17–2895.45	1670.79	2301.90	72.58
51–70 years, female	51	2641–9633	5843.84 (1466.42)	631.82–2304.55	1398.05	1974.18	70.82
51–70 years, male	20	3391–13,265	6870.30 (2697.48)	811.24–3173.44	1643.61	2301.90	71.40
Above 70 years, female	10	2866–7551	5443.20 (1483.89)	685.65–1806.46	1302.20	1974.18	65.96
Above 70 years, male	5	3525–9102	6146.60 (2200.72)	843.30–2177.51	1470.48	2301.90	63.88

^a^ EER: estimated energy requirement; ^b^ kJ: Kilojoules; ^c^ SD: Standard deviation; ^d^ kCal: Kilocalories; ^e^ Institute of Medicine 2005; Average values were calculated using sedentary physical activity levels for each of the age groups and the minimum and maximum EER values for normal BMI were used to calculate the average EER for those above 19 years; ^f^ Minimum energy intake; ^g^ Maximum energy intake.

**Table 9:** The mean frequency scores ^1^ for the ten most commonly consumed food items.

Food Items	N ^2^	Mean	Food Items	n	Mean
Oil	156	3.37	Tomato	157	2.29
Onions	159	3.31	Mayonnaise	159	2.28
*Phutu*	158	2.63	Rice, white	156	2.15
Sweets	151	2.35	Apple, unpeeled medium	159	2.13
Brown bread/roll	145	2.33	Eggs	158	2.12

^1^ The average mean frequency score was calculated using the number of days a household consumed a specific food item in a month; ^2^ n: Indicates the number of households that consumed that particular food item.

## Data Availability

The data presented in this study are available on request from the corresponding author.

## Supplementary Materials

The following are available online at https://www.mdpi.com/article/10.3390/nu13092920/s1, Table S1: EAR/AI values used to compare the mean nutrient intake from two 24 h repeated recalls for individuals aged 1–18 years; Table S2: EAR/AI values used to compare the mean nutrient intake from two 24 h repeated recalls for individuals aged 19–70 years; Table S3: The prevalence of adequate and inadequate nutrient intake for each age and gender group; Table S4: Results of the food frequency analysis; Table S5: The mean frequency scores for commonly consumed food items.

## References

[1] Development Intiatives (2017). Nourishing the SDGs: Global Nutrition Report 2017.

[2] Food and Agriculture Organization (FAO), International Fund for Agricultural Development (IFAD), United Nations Children’s Fund (UNICEF), World Food Programme (WFP), World Health Organization (WHO) (2017). The State of Food Security and Nutrition in the World 2017. Building Resilience for Peace and Food Security.

[3] DevelopmentInitiatives (2020). 2020 Global Nutrition Report: Action on Equity to End Malnutrition.

[4] DevelopmentInitiatives Global Nutrition Report 2018: Shining a Light to Spur Action on Nutrition. https://globalnutritionreport.org/reports/global-nutrition-report-2018.

[5] Shisana O., Labadarios D., Rehle T., Simbayi L., Zuma K., Dhansay A., Reddy P., Parker W., Hoosain E., Naidoo P. (2013). South African National Health and Nutrition Examination Survey (SANHANES-1).

[6] National Department of Health (nDoH), Statistics South Africa (Stats SA), South African Medical Research Council (SAMRC), ICF (2017). South Africa Demographic and Health Survey 2016: Key Indicators.

[7] Labadarios D., Moodie I., Rensburg A. (2007). Vitamin A Status: In Labadarios D. National Food Consumption Survey: Fortification Baseline, Chapter 9B: South Africa 2005.

[8] Labadarios D., Steyn N., Maunder E., Macintyre U., Swart R., Gericke G., Huskisson J., Dannhauser A., Vorster H., Nesamvumi E. (2000). The National Food Consumption Survey (NFCS): Children Aged 1–9 years, South Africa, 1999.

[9] Labadarios D., Middelkoop A.V. (1996). The South African Vitamin A Consultative Group. Children Aged 6–71 Months in South Africa, 1994; Their Anthropometric, Vitamin A, Iron and Immunization Coverage Status. S. Afr. Med. J..

[10] United Nations Children’s Fund (UNICEF), World Health Organization (WHO), World Bank Group Levels and Trends in Child Malnutrition. http://data.unicef.org/topic/nutrition/malnutrition/.

[11] United Nations Children’s Fund (UNICEF) Stunting Reflects Chronic Undernutrition During the Most Critical Periods of Growth and Development in Early Life. http://unicef.in/Whatwedo/10/stunting.

[12] Faber M., Wenhold F. (2007). Nutrition in contemporary South Africa. Water SA.

[13] Bain L.E., Awah P.K., Geraldine N., Kindong N.P., Siga Y., Bernard N., Tanjeko A.T. (2013). Malnutrition in Sub–Saharan Africa: Burden, causes and prospects. Pan Afr. Med. J..

[14] Kimani-Murage E.W., Kahn K., Pettifor J.M., Tollman S.M., Dunger D.B., Gómez-Olivé X.F., Norris S.A. (2010). The prevalence of stunting, overweight and obesity, and metabolic disease risk in rural South African children. BMC Public Health.

[15] Chopra M., Daviaud E., Pattinson R., Fonn S., Lawn J.E. (2009). Saving the lives of South Africa’s mothers, babies, and children: Can the health system deliver?. Lancet.

[16] Manary M.J., Sandige H.L. (2008). Management of acute moderate and severe childhood malnutrition. BMJ.

[17] Smuts C.M., Faber M., Schoeman S.E., Laubscher J.A., Oelofse A., Benade A.S., Dhansay M. (2008). Socio-demographic factors and anthropometric status of 0–71-month-old children and their caregivers in rural districts of the Eastern Cape and KwaZulu-Natal provinces of South Africa. S. Afr. J. Clin. Nutr..

[18] Escott-Stump S. (2015). Nutrition and Diagnosis-Related Care.

[19] Joubert J., Norman R., Bradshaw D., Goedecke J.H., Steyn N.P., Puoane T. (2007). Estimating the burden of disease attributable to excess body weight in South Africa in 2000. S. Afr. Med. J..

[20] Labadarios D., Swart R., Maunder E., Kruger H., Gericke G., Kuzwayo P., Ntsie P., Steyn N., Schloss I., Dhansay M. (2008). The National Food Consumption Survey Fortification Baseline (NFCS-FB). S. Afr. J.Clin. Nutr..

[21] Armstrong M., Lambert M., Sharwood K., Lambert E. (2006). Obesity and overweight in South African primary school children—the Health of the Nation Study. J. Endocrinol. Metab. Diabetes S. Afr..

[22] Sizer F., Whitney E. (2017). Nutrition Concepts and Controversies.

[23] Lutter C.K., Daelmans B.M., de Onis M., Kothari M.T., Ruel M.T., Arimond M., Deitchler M., Dewey K.G., Blössner M., Borghi E. (2011). Undernutrition, poor feeding practices, and low coverage of key nutrition interventions. Pediatrics.

[24] Schönfeldt H., Gibson N., Vermeulen H. (2010). News and views: The possible impact of inflation on nutritionally vulnerable households in a developing country using South Africa as a case study. Nutr. Bull..

[25] World Health Organization (WHO) Malnutrition: Maternal, New Born, Child and Adolescent Health. http://www.who.int/maternal_child_adolescent/topics/child/malnutrition/en/index.html.

[26] Statistics South Africa (Stats SA) (2017). Poverty Trends in South Africa. An Examination of Absolute Poverty between 2016 and 2015.

[27] Argent J., Finn A., Leibbrandt M., Wooland I. (2009). NIDS National Income Dynamics Study; Poverty: Analysis of the NIDS Wave 1 Dataset Discussion Paper no.13. http://www.nids.uct.ac.za/home/index.php?/Nids-Documentation/discussion-papers.html.

[28] Statistics South Africa (Stats SA) General Household Survey 2018. http://www.statssa.gov.za/publications/P0318/P03182018.pdf.

[29] Wenhold F., Annandale J., Faber M., Hart T. (2012). Water Use and Nutrient Content of Crop and Animal Food Products for Improved Household Food Security: A Scoping Study: Report to the Water Research Commission TT 537/12.

[30] Mabhaudhi T., Chibarabada T., Modi A. (2016). Water-food-nutrition-health nexus: Linking water to improving food, nutrition and health in Sub-Saharan Africa. Int. J. Environ. Res. Public Health.

[31] RepublicofSouthAfrica (RSA) Tito Titus Mboweni Minister of Finance: 2021 Budget Speech. http://www.treasury.gov.za/documents/National%20Budget/2021/speech/speech.pdf.

[32] Republicof South Africa (RSA) Tito Titus Mboweni Minister of Finance: 2019 Budget Speech. http://www.treasury.gov.za/documents/national%20budget/2019/speech/speech.pdf.

[33] National Agricultural Marketing Council (NAMC) Markets and Economic Research Centre: Food Basket Price Monthly. https://www.namc.co.za/wp-content/uploads/2021/03/Food-Basket-March-2021.pdf.

[34] World Health Organization (WHO) Global Database on Child Growth and Malnutrition. http://www.int/nutgrowthdb/about/introduction/en/index5.html.

[35] Lee R.D., Lee R.D., Nieman D.C. (2013). Nutritional Assessment.

[36] WHO (2008). Training Course on Child Growth Assessment.

[37] WHO Integrated Management of Childhood Illness (IMAM): Distance Learning Course Module 6; Malnutrition and Anaemia. http://apps.who.int/iris/bitstream/10665/104772/8/9789241506823_Module6_eng.pdf?ua=1.

[38] Gibson R. (2005). Principles of Nutritional Assessment.

[39] WHO (2008). Waist Circumference and Waist-Hip Ratio. Report of a WHO Expert Consultation Geneva, 8–11 December 2008.

[40] Fagúndez L., Torres A., Sánchez M., Aured M.d.T., Rodrigo C., Rocamora J. (2015). Diet history: Method and applications. Nutr. Hosp..

[41] Sheehy T., Kolahdooz F., Mtshali T., Khamis T., Sharma S. (2014). Development of a quantitative food frequency questionnaire for use among rural S outh A fricans in K wa Z ulu-N atal. J. Hum. Nutr. Diet..

[42] Faber M., Laubscher R., Laurie S. (2013). Availability of, access to and consumption of fruits and vegetables in a peri-urban area in KwaZulu-Natal, South Africa. Matern. Child Nutr..

[43] Faber M., Kruger H.S. (2005). Dietary intake, perceptions regarding body weight, and attitudes toward weight control of normal weight, overweight, and obese black females in a rural village in South Africa. Ethn. Dis..

[44] Spearing K., Kolahdooz F., Lukasewich M., Mathe N., Khamis T., Sharma S. (2013). Nutritional composition of commonly consumed composite dishes from rural villages in Empangeni, Kwa Zulu-Natal, South Africa. J. Hum. Nutr. Diet..

[45] Rodrigo C.P., Aranceta J., Salvador G., Varela-Moreiras G. (2015). Food frequency questionnaires. Nutr. Hosp..

[46] Coulston A., Boushey C., MGFerruzzi (2013). Nutrition in the Prevention and Treatment of Disease.

[47] Department of Health (DoH) (2018). Standard Operating Procedures on the Prevention and Management of Malnutrition in KZN.

[48] Institue of Medicine (2006). Dietary DRI Refernce Intakes: The Essential Guide to Nutrient Requirement.

[49] Murphy S.P., Guenther P.M., Kretsch M.J. (2006). Using the dietary reference intakes to assess intakes of groups: Pitfalls to avoid. J. Acad. Nutr. Diet..

[50] Institue of Medicine (2001). Dietary Reference Intake for Vitamin A, Vitamin K, Arsenic, Boron, Chromium, Copper, Iodine, Iron, Manganese, Molybdenum, Nickel, Silicon, Vanadium and Zinc.

[51] Institue of Medicine (2001). Dietary Reference Intakes: Application in Dietary Assessment. Food and Nutrition Board.

[52] Schoeman S., Faber M., Adams V., Smuts C., Ford-Ngomane N., Laubscher J., Dhansay M. (2010). Adverse social, nutrition and health conditions in rural districts of KwaZulu-Natal and the Eastern Cape provinces, South Africa. S. Afr. J. Clin. Nutr..

[53] Danaei G., Andrews K.G., Sudfeld C.R., Fink G., McCoy D.C., Peet E., Sania A., Smith Fawzi M.C., Ezzati M., Fawzi W.W. (2016). Risk factors for childhood stunting in 137 developing countries: A comparative risk assessment analysis at global, regional, and country levels. PLoS Med..

[54] Health Systems Trust (HST) Malnutrition Causes, Economic Loss, Half of All Child Deaths. http://www.hst.org.za/news/malnutrition-causes-economic-loss-half-all-child-deaths.

[55] Omidire M.F., AnnaMosia D., Mampane M.R. (2015). Perceptions of the roles and responsibilities of caregivers in children’s homes in South Africa. Rev. Asistenta Soc..

[56] Bonnell E.K., Huggins C.E., Huggins C.T., McCaffrey T.A., Palermo C., Bonham M.P. (2017). Influences on dietary choices during day versus night shift in shift workers: A mixed methods study. Nutrients.

[57] Kamphuis C.B., de Bekker-Grob E.W., van Lenthe F.J. (2015). Factors affecting food choices of older adults from high and low socioeconomic groups: A discrete choice experiment. Am. J. Clin. Nutr..

[58] Kearney J. (2010). Food consumption trends and drivers. Philos. Trans. R. Soc. B Biol. Sci..

[59] Govender L., Pillay K., Derera J., Siwela M. (2014). Acceptance of a complementary food prepared with yellow, provitamin A-biofortified maize by black caregivers in rural KwaZulu-Natal. S. Afr. J. Clin. Nutr..

[60] Senekal M., Steyn N.P., Nel J.H. (2003). Factors associated with overweight/obesity in economically active South African populations. Ethn. Dis..

[61] Puoane T., Steyn K., Bradshaw D., Laubscher R., Fourie J., Lambert V., Mbananga N. (2002). Obesity in South Africa: The South African demographic and health survey. Obes. Res..

[62] Nyberg S.T., Batty G.D., Pentti J., Virtanen M., Alfredsson L., Fransson E.I., Goldberg M., Heikkilä K., Jokela M., Knutsson A. (2018). Obesity and loss of disease-free years owing to major non-communicable diseases: A multicohort study. Lancet Public Health.

[63] World Health Organization (WHO) Global Status Report on Noncommunicable Diseases 2010. www.who.intlnmhlpublication/ncd_report_full_en.pdf.

[64] Hawkins S., Oken E., Gillman M., Halfon N., Forrest C., Lerner R., Faustma E. (2018). Early in the life course: Time for obesity prevention. Handbook if Life Course Health Development.

[65] Zhu S., Heshka S., Wang Z., Shen W., Allison D.B., Ross R., Heymsfield S.B. (2004). Combination of BMI and waist circumference for identifying cardiovascular risk factors in whites. Obes. Res..

[66] Gierach M., Gierach J., Ewertowska M., Arndt A., Junik R. (2014). Correlation between body mass index and waist circumference in patients with metabolic syndrome. ISRN Endocrinol..

[67] Wildman R.P., Gu D., Reynolds K., Duan X., Wu X., He J. (2005). Are waist circumference and body mass index independently associated with cardiovascular disease risk in Chinese adults?. Am. J. Clin. Nutr..

[68] Janssen I., Heymsfield S.B., Allison D.B., Kotler D.P., Ross R. (2002). Body mass index and waist circumference independently contribute to the prediction of nonabdominal, abdominal subcutaneous, and visceral fat. Am. J. Clin. Nutr..

[69] Abu-Ouf N.M., Jan M.M. (2015). The impact of maternal iron deficiency and iron deficiency anemia on child’s health. Saudi Med. J..

[70] Duncan P., Howe L., Manukusa Z., Purdy S. (2014). Determinants of obesity and perception of weight in hypertensive patients in rural South Africa. S. Afr. J. Clin. Nutr..

[71] Devanathan R., Esterhuizen T.M., Govender R.D. (2013). Overweight and obesity amongst Black women in Durban, KwaZulu-Natal: A ‘disease’of perception in an area of high HIV prevalence. Afr. J. Prim. Health Care Fam. Med..

[72] Okop K.J., Mukumbang F.C., Mathole T., Levitt N., Puoane T. (2016). Perceptions of body size, obesity threat and the willingness to lose weight among black South African adults: A qualitative study. BMC Public Health.

[73] Ziraba A.K., Fotso J.C., Ochako R. (2009). Overweight and obesity in urban Africa: A problem of the rich or the poor?. BMC Public Health.

[74] World Health Organization (WHO) (2011). Guideline: Vitamin A Supplementation in Infants and Children 6–59 Months of Age.

[75] Swart R., Sanders D., McLachlan M. (2008). Nutrition: A primary health care perspective: Primary health care: Programme areas. South. African Health Review.

[76] Department of Health (DoH), United Nations Children’s Fund (UNICEF) (2007). A Reflection of the South African Maize Meal and Wheat Flour Fortification Programme (2004 to 2007).

[77] Castell G.S., Serra-Majem L., Ribas-Barba L. (2015). What and how much do we eat? 24-hour dietary recall method. Nutr. Hosp..

[78] Nel J.H., Casey A. (2003). Secondary data analyses of dietary surveys undertaken in South Africa to determine usual food consumption of the population. Public Health Nutr..

[79] Faber M., van Jaarsveld P.J., Kunneke E., Kruger H.S., Schoeman S.E., van Stuijvenberg M.E. (2015). Vitamin A and anthropometric status of South African preschool children from four areas with known distinct eating patterns. Nutrition.

[80] Li Y., Hruby A., Bernstein A.M., Ley S.H., Wang D.D., Chiuve S.E., Sampson L., Rexrode K.M., Rimm E.B., Willett W.C. (2015). Saturated fats compared with unsaturated fats and sources of carbohydrates in relation to risk of coronary heart disease: A prospective cohort study. J. Am. Coll. Cardiol..

[81] López-Alarcón M., Perichart-Perera O., Flores-Huerta S., Inda-Icaza P., Rodríguez-Cruz M., Armenta-Álvarez A., Bram-Falcón M.T., Mayorga-Ochoa M. (2014). Excessive refined carbohydrates and scarce micronutrients intakes increase inflammatory mediators and insulin resistance in prepubertal and pubertal obese children independently of obesity. Mediat. Inflamm..

[82] Fabbri A.D., Crosby G.A. (2016). A review of the impact of preparation and cooking on the nutritional quality of vegetables and legumes. Int. J. Gastron. Food Sci..

[83] Wiklund P. (2016). The role of physical activity and exercise in obesity and weight management: Time for critical appraisal. J. Sport Health Sci..

[84] Phatlhane D.V., Zemlin A.E., Matsha T.E., Hoffmann M., Naidoo N., Ichihara K., Smit F., Erasmus R.T. (2016). The iron status of a healthy South African adult population. Clin. Chim. Acta.

[85] King J.C. (2016). A summary of pathways or mechanisms linking preconception maternal nutrition with birth outcomes. J. Nutr..

[86] He K., Hu F., Colditz G., Manson J., Willett W., Liu S. (2004). Changes in intake of fruits and vegetables in relation to risk of obesity and weight gain among middle-aged women. Int. J. Obes..

[87] Steinemann N., Grize L., Ziesemer K., Kauf P., Probst-Hensch N., Brombach C. (2017). Relative validation of a food frequency questionnaire to estimate food intake in an adult population. Food Nutr. Res..

