# Supplementary material for: Assessment of the Nutritional Status of Four Selected Rural Communities in KwaZulu-Natal, South Africa
Source: Nutrients. 2021 Aug 24;13(9):2920. doi: 10.3390/nu13092920 (PMC8465248; doi:10.3390/nu13092920)
Supplement: Supplementary file 1 [file nutrients-13-02920-s001.zip › nutrients-1299211-supplementary.pdf]

Table S1. EAR/AI values used to compare the mean nutrient intake from two 24-hour repeated recalls for individuals aged 1-18 years

Nutrients	1-3 years		4-8 years		9-13 years, Female		9-13 years, Male		14-18 years, Female		14-18 years, Male	
	EAR[a-d]	AI[e]	EAR	AI	EAR	AI	EAR	AI	EAR	AI	EAR	AI
**Total protein (g)**	11	-	15	-	28	-	27	-	28	-	44	-
**Carbohydrates (g)**	100	-	100	-	100	-	100	-	100	-	100	-
**Dietary fibre (g)**	19	-	25	-	26	-	31	-	26		38	-
**Calcium (mg)**		500		800	-	1300		1300	-	1300	-	1300
**Magnesium (mg)**	65	-	110	-	200	-	200	-	300	-	340	-
**Phosphorus (mg)**	380	-	405	-	1055	-	1055	-	1055	-	1055	-
**Iron (mg)**	3	-	4.1	-	5.7	-	5.9	-	7.9	-	7.7	-
**Zinc (mg)**	2.5	-	4.0	-	7.0	-	7.0	-	7.3	-	8.5	-
**Thiamine (mg)**	0.4	-	0.5	-	0.7	-	0.7	-	0.9	-	1.0	-
**Riboflavin (mg)**	0.4	-	0.5	-	0.8	-	0.8	-	0.9	-	1.1	-
**Niacin (mg)**	5	-	6	-	9	-	9	-	11	-	12	-
**Vitamin B6 (mg)**	0.4	-	0.5	-	0.8	-	0.8	-	1.0	-	1.1	-
**Folate (μg)**	120	-	160	-	250	-	250	-	330	-	330	-
**Vitamin B12 (μg)**	0.7	-	1	-	1.5	-	1.5	-	1.5	-	2.0	-
**Vitamin C (mg)**	13	-	22	-	39	-	39	-	56	-	63	-
**Vitamin A (μg)**	210	-	275	-	420	-	445	-	485	-	630	-
**Vitamin D (μg)**	-	5.0		5.0	-	5.0		5.0	-	5.0	-	5.0
**Vitamin E (mg)**	5	-	6	-	9	-	9	-	12	-	12	-
**Vitamin K (μg)**	30	-	55	-	60	-	60	-	75	-	75	-

[a] Institute of Medicine 2003; [b]Institute of Medicine 2001b; [c]Institute of Medicine 2000; [d]Institute of Medicine 1998; [e]Institute of Medicine 1997; SD, standard deviation; EAR, estimated average requirement; AI, adequate intake; * p values given in bold font indicate that the mean nutrient intake is significantly different from the EAR/AI.

Table S2. EAR/AI values used to compare the mean nutrient intake from two 24-hour repeated recalls for individuals aged 19-70 years

Nutrients	19-30 years, Female		19-30 years, Male		31-50 years, Female		31-50 years, Male		51-70 years, Female		51-70 years, Male	
	EAR[a-d]	AI[e]	EAR	AI	EAR	AI	EAR	AI	EAR	AI	EAR	AI
**Total protein (g)**	38	-	46	-	38	-	46	-	38	-	46	-
**Carbohydrates (g)**	100	-	100	-	100	-	100	-	100	-	100	-
**Dietary fibre (g)**	25	-	38	-	25	-	38	-	25	-	38	-
**Calcium (mg)**	-	1000	-	1000	-	1000	-	1000	-	1000	-	1000
**Magnesium (mg)**	255	-	330	-	265	-	350	-	255	-	330	-
**Phosphorus (mg)**	580	-	580	-	580	-	580	-	580	-	580	-
**Iron (mg)**	8.1	-	6	-	8.1	-	6	-	8.1	-	6	-
**Zinc (mg)**	6.8	-	9.4	-	6.8	-	9.4	-	6.8	-	9.4	-
**Thiamine (mg)**	0.9	-	1.0	-	0.9	-	1.0	-	0.9	-	1.0	-
**Riboflavin (mg)**	0.9	-	1.1	-	0.9	-	1.1	-	0.9	-	1.1	-
**Niacin (mg)**	11	-	12	-	11	-	12	-	11	-	12	-
**Vitamin B6 (mg)**	1.1	-	1.1	-	1.1	-	1.1	-	1.1	-	1.1	-
**Folate (µg)**	320	-	320	-	320	-	320	-	320	-	320	-
**Vitamin B12 (µg)**	2.0	-	2.0	-	2.0	-	2.0	-	2.0	-	2.0	-
**Vitamin C (mg)**	60	-	75	-	60	-	75	-	60	-	75	-
**Vitamin A (µg)**	500	-	625	-	500	-	625	-	500	-	625	-
**Vitamin D (µg)**	-	5.0	-	5.0	-	5.0	-	5.0		10	-	10
**Vitamin E (mg)**	12	-	12	-	12	-	12	-	12	-	12	-
**Vitamin K (µg)**	90	-	120	-	90	-	120	-	90	-	120	-

[a] Institute of Medicine 2003; [b]Institute of Medicine 2001b; [c]Institute of Medicine 2000; [d]Institute of Medicine 1998; [e]Institute of Medicine 1997; SD, standard deviation; EAR, estimated average requirement; AI, adequate intake; * p values given in bold font indicate that the mean nutrient intake is significantly different from the EAR/AI.

Table S3. The prevalence of adequate and inadequate nutrient intake for each age and gender group

Age group (yrs)	n	Gender	Nutrients with adequate intake
1-3	16	Male and female	Protein, carbohydrates, magnesium, phosphorus, iron, zinc, thiamine, vitamin B6, B12 and C.
4-8	59	Male and female	Protein, carbohydrates, magnesium, phosphorus, iron, thiamine, niacin and vitamin B6.
9-13	37	Female	Protein, carbohydrates, iron, thiamine, niacin, vitamin B6 and B12.
9-13	14	Male	Protein, carbohydrates, magnesium, iron, thiamine, vitamin B6 and B12.
14-18	32	Female	Protein, carbohydrate, vitamin B6 and B12.
14-18	18	Male	Carbohydrates
19-30	67	Female	Protein, carbohydrates, phosphorus and niacin.
19-30	43	Male	Protein, carbohydrates, phosphorous and iron.
31-50	62	Female	Protein, carbohydrates, phosphorus, zinc, thiamine and niacin.
31-50	32	Male	Protein, carbohydrates, iron and niacin.
51-70	51	Female	Carbohydrates and phosphorus.
51-70	20	Male	Protein, carbohydrates, magnesium and niacin.
Above 70	10	Female	Protein, carbohydrates, phosphorus and vitamin K.
Above 70	5	Male	Carbohydrates, phosphorus and niacin.
**Age group (yrs)**	**n**	**Gender**	**Nutrients with inadequate intake**
1-3	16	Male and female	Dietary fibre, riboflavin, niacin, folate, vitamins A, E and K.
4-8	59	Male and female	Dietary fibre, zinc, riboflavin, folate, vitamins B_{12}, vitamin C, A, E and K.
9-13	37	Female	Dietary fibre, magnesium, phosphorous, zinc, riboflavin, folate, vitamins C, A, E and K.
9-13	14	Male	Dietary fibre, phosphorus, zinc, riboflavin, niacin, folate, vitamins C, A, E and K.
14-18	32	Female	Dietary fibre, magnesium, phosphorous, iron, zinc, thiamine, riboflavin, niacin, folate, vitamins C, A, E and K.
14-18	18	Male	Total protein, dietary fibre, magnesium, phosphorous, iron, zinc, thiamine, riboflavin, niacin, folate, vitamins B_6, B_{12}, C, A, E and K.
19-30	67	Female	Dietary fibre, magnesium, iron, zinc, thiamine, riboflavin, folate, vitamins B_6, B_{12}, C, A, E and K.
19-30	43	Male	Dietary fibre, magnesium, zinc, thiamine, riboflavin, niacin, folate, vitamins B_6, B_{12}, C, A, E and K.
31-50	62	Female	Dietary fibre, magnesium, iron, riboflavin, folate, vitamins B_6, B_{12}, C, A, E and K.
31-50	32	Male	Dietary fibre, magnesium, phosphorous, zinc, thiamine, riboflavin, folate, vitamins B_6, B_{12}, C, A, E and K.
51-70	51	Female	Dietary fibre, magnesium, iron, zinc, thiamine, riboflavin, niacin, folate, vitamins B_6, B_{12}, C, A, E and K.
51-70	20	Male	Dietary fibre, magnesium, iron, zinc, thiamine, riboflavin, folate, vitamins B_6, B_{12}, C, A, E and K.
Above 70	10	Female	Dietary fibre, magnesium, iron, zinc, thiamine, riboflavin, niacin, folate, vitamins B_6, B_{12}, C, A and E.
Above 70	5	Male	Total protein, dietary fibre, magnesium, iron, zinc, thiamine, riboflavin, folate, vitamins B_6, B_{12}, C, A, E and K.

Table S4. Results of the food frequency analysis[1]

Food Item	Never/ less than once a month		1-3 times per month		Once a week		2-4 times per week		5-6 times per week		7 times per week		2-3 times per day		4-5 times per day		>6 times per day	
	**n**	**%**	**n**	**%**	**n**	**%**	**n**	**%**	**n**	**%**	**n**	**%**	**n**	**%**	**n**	**%**	**n**	**%**
**Cereals and grains**																		
Cornflakes	71	44.4	20	12.5	19	11.9	29	18.1	4	2.5	15	9.4	1	0.6	1	0.6	0	0.0
Future life	106	74.1	16	11.2	11	7.7	4	2.8	2	1.4	3	2.1	1	0.7	0	0.0	0	0.0
Mageu	57	35.8	20	12.6	24	15.1	31	19.5	16	10.1	5	3.1	4	2.5	1	0.6	1	0.6
Maize meal, stiff	47	29.7	16	10.1	35	22.2	36	22.8	12	7.6	9	5.7	3	1.9	0	0.0	0	0.0
Maize meal porridge	62	39.5	30	19.1	27	17.2	24	15.3	8	5.1	3	1.9	3	1.9	0	0.0	0	0.0
Maltabella	116	73.9	14	8.9	9	5.7	10	6.4	2	1.3	6	3.8	0	0.0	0	0.0	0	0.0
Oats	130	82.8	6	3.8	13	8.3	6	3.8	1	0.6	1	0.6	0	0.0	0	0.0	0	0.0
Pasta	107	66.9	12	7.5	5	3.1	17	10.6	13	8.1	4	2.5	2	1.3	0	0.0	0	0.0
Phutu	42	26.6	5	3.2	12	7.6	44	27.8	39	24.7	2	1.3	10	6.3	3	1.9	1	0.6
Pronutro	136	87.2	4	2.6	6	3.8	3	1.9	5	3.2	0	0.0	2	1.3	0	0.0	0	0.0
Rice, brown	108	68.4	4	2.5	6	3.8	27	17.1	10	6.3	1	0.6	0	0.0	1	0.6	1	0.6
Rice, white	37	23.7	17	10.9	26	16.7	52	33.3	15	9.6	4	2.6	4	2.6	1	0.6	0	0.0
Samp and beans	50	31.3	43	26.9	43	26.9	15	9.4	3	1.9	4	2.5	1	0.6	1	0.6	0	0.0
Weetbix	73	47.1	13	8.4	20	12.9	23	14.8	15	9.7	10	6.5	1	0.6	0	0.0	0	0.0
**Bread**																		
Brown bread/roll	29	20.0	21	14.5	20	13.8	42	29.0	17	11.7	13	9.0	3	2.1	0	0.0	0	0.0
Ujeqe	46	46.5	8	8.1	21	21.2	15	15.2	8	8.1	0	0.0	1	1.0	0	0.0	0	0.0
White bread/roll	45	28.7	24	15.3	25	15.9	29	18.5	20	12.7	12	7.6	2	1.3	0	0.0	0	0.0
**Biscuits and snacks**																		
Biscuit, filling	67	44.7	40	26.7	26	17.3	12	8.0	2	1.3	2	1.3	1	0.7	0	0.0	0	0.0
Biscuit	71	44.4	42	26.3	23	14.4	11	6.9	7	4.4	4	2.5	1	0.6	0	0.0	1	0.6
Cream crackers	112	70.0	27	16.9	9	5.6	5	3.1	2	1.3	2	1.3	2	1.3	0	0.0	1	0.6
**Starchy vegetables**																		

[1] The researcher had prior knowledge that bambara groundnut was unfamiliar and not commonly consumed by the individuals residing in the study sites investigated. However, this is a limitation to the study and bambara groundnut should have been included in the FFQ.

Table S4. Results of the food frequency analysis continued

	Never/ less than once a month		1-3 times per month		Once a week		2-4 times per week		5-6 times per week		7 times per week		2-3 times per day		4-5 times per day		>6 times per day	
	**n**	**%**	**n**	**%**	**n**	**%**	**n**	**%**	**n**	**%**	**n**	**%**	**n**	**%**	**n**	**%**	**n**	**%**
Amadumbe	52	32.7	47	29.6	22	13.8	18	11.3	8	5.0	4	2.5	4	2.5	1	0.6	3	1.9
Mealie/corn (on cob)	49	31.4	33	21.2	28	17.9	33	21.2	4	2.6	3	1.9	6	3.8	0	0.0	0	0.0
Peas, green	102	65.0	22	14.0	14	8.9	14	8.9	2	1.3	1	0.6	0	0.0	0	0.0	1	0.6
Potato, mashed, boiled, pumpkin, winter squash	37	23.3	28	17.6	35	22.0	45	28.3	7	4.4	3	1.9	3	1.9	0	0.0	1	0.6
Sweet potato	34	21.7	44	28.0	41	26.1	29	18.5	5	3.2	3	1.9	1	0.6	0	0.0	0	0.0
**Starchy foods prepared with fats**																		
Fried chips	54	34.2	31	19.6	33	20.9	28	17.7	5	3.2	6	3.8	1	0.6	0	0.0	0	0.0
Instant noodles	82	51.9	23	14.6	22	13.9	17	10.8	4	2.5	6	3.8	4	2.5	0	0.0	0	0.0
Popcorn with oil	95	60.1	31	19.6	16	10.1	14	8.9	2	1.3	0	0.0	0	0.0	0	0.0	0	0.0
Scone	57	35.8	57	35.8	23	14.5	12	7.5	4	2.5	4	2.5	1	0.6	1	0.6	0	0.0
Vetkoek	37	25.7	54	37.5	23	16.0	17	11.8	8	5.6	2	1.4	3	2.1	0	0.0	0	0.0
**Fruit**																		
Apple, unpeeled medium	29	18.2	38	23.9	30	18.9	35	22.0	7	4.4	12	7.5	8	5.0	0	0.0	0	0.0
Banana, small	37	23.4	26	16.5	49	31.0	29	18.4	8	5.1	7	4.4	2	1.3	0	0.0	0	0.0
Canned fruit	75	47.2	46	28.9	15	9.4	14	8.8	3	1.9	4	2.5	1	0.6	0	0.0	1	0.6
Fruit juice	58	36.5	30	18.9	25	15.7	18	11.3	9	5.7	9	5.7	10	6.3	0	0.0	0	0.0
Fruit salad, fresh	82	51.6	43	27.0	20	12.6	4	2.5	3	1.9	3	1.9	2	1.3	1	0.6	1	0.6
Grapes, small	52	33.1	63	40.1	21	13.4	6	3.8	4	2.5	8	5.1	2	1.3	0	0.0	1	0.6
Orange	38	24.4	44	28.2	31	19.9	24	15.4	4	2.6	8	5.1	5	3.2	1	0.6	1	0.6
Pear	73	46.8	40	25.6	19	12.2	15	9.6	2	1.3	4	2.6	2	1.3	1	0.6	0	0.0
Guava	9	81.8	1	9.1	0	0.0	0	0.0	0	0.0	1	9.1	0	0.0	0	0.0	0	0.0
**Milk and milk products**																		
Custard sweetened full cream	98	62.4	34	21.7	16	10.2	3	1.9	2	1.3	2	1.3	1	0.6	1	0.6	0	0.0
Custard Ultra Mel	70	44.0	42	26.4	29	18.2	7	4.4	5	3.1	4	2.5	1	0.6	1	0.6	0	0.0
Full cream milk	62	38.8	26	16.3	14	8.8	24	15.0	13	8.1	7	4.4	9	5.6	2	1.3	3	1.9
Low fat milk	92	57.5	26	16.3	10	6.3	16	10.0	8	5.0	5	3.1	2	1.3	0	0.0	1	0.6

Table S4. Results of the food frequency analysis continued

	Never/ less than once a month		1-3 times per month		Once a week		2-4 times per week		5-6 times per week		7 times per week		2-3 times per day		4-5 times per day		>6 times per day	
	n	%	n	%	n	%	n	%	n	%	n	%	n	%	n	%	n	%
Maas	52	33.3	30	19.2	24	15.4	31	19.9	8	5.1	4	2.6	4	2.6	0	0.0	3	1.9
Yoghurt flavoured	82	52.2	34	21.7	26	16.6	9	5.7	0	0.0	3	1.9	2	1.3	0	0.0	1	0.6
Yoghurt low fat	120	75.9	17	10.8	11	7.0	7	4.4	2	1.3	1	0.6	0	0.0	0	0.0	0	0.0
Yoghurt plain	123	77.8	20	12.7	12	7.6	1	0.6	1	0.6	1	0.6	0	0.0	0	0.0	0	0.0
**Vegetables**																		
Beans, green	97	61.4	17	10.8	29	18.4	14	8.9	0	0.0	1	0.6	0	0.0	0	0.0	0	0.0
Beetroot	73	46.8	36	23.1	29	18.6	9	5.8	6	3.8	0	0.0	2	1.3	0	0.0	1	0.6
Broccoli	113	71.1	18	11.3	15	9.4	9	5.7	3	1.9	0	0.0	1	0.6	0	0.0	0	0.0
Cabbage	47	29.2	24	14.9	44	27.3	31	19.3	11	6.8	2	1.2	2	1.2	0	0.0	0	0.0
Cauliflower	132	82.5	11	6.9	10	6.3	4	2.5	0	0.0	3	1.9	0	0.0	0	0.0	0	0.0
Imifino (Indigenous leafy vegetables)	10	43.5	4	17.4	4	17.4	5	21.7	0	0.0	0	0.0	0	0.0	0	0.0	0	0.0
Lettuce	104	65.0	19	11.9	16	10.0	8	5.0	6	3.8	1	0.6	5	3.1	1	0.6	0	0.0
Mixed vegetable	68	42.5	15	9.4	15	9.4	26	16.3	17	10.6	7	4.4	11	6.9	0	0.0	1	0.6
Mushroom	116	73.4	19	12.0	7	4.4	9	5.7	3	1.9	2	1.3	1	0.6	0	0.0	1	0.6
Peppers	72	45.3	12	7.5	10	6.3	14	8.8	30	18.9	8	5.0	8	5.0	2	1.3	3	1.9
Onions	25	15.7	18	11.3	11	6.9	16	10.1	54	34.0	8	5.0	16	10.1	2	1.3	9	5.7
Spinach	34	21.9	38	24.5	41	26.5	25	16.1	9	5.8	2	1.3	3	1.9	1	0.6	2	1.3
Tomato	26	16.6	29	18.5	27	17.2	43	27.4	20	12.7	6	3.8	4	2.5	2	1.3	0	0.0
**Meat and meat substitutes**																		
Baked beans	56	35.0	27	16.9	37	23.1	30	18.8	3	1.9	5	3.1	0	0.0	0	0.0	1	0.6
Beans, lentils and peas	87	54.4	31	19.4	26	16.3	12	7.5	1	0.6	1	0.6	2	1.3	0	0.0	0	0.0
Bean salad, no oil	85	53.5	28	17.6	37	23.3	7	4.4	0	0.0	0	0.0	2	1.3	0	0.0	0	0.0
Beef, cuts	46	28.6	35	21.7	41	25.5	26	16.1	6	3.7	6	3.7	1	0.6	0	0.0	0	0.0
Beef, mince	84	52.8	34	21.4	26	16.4	12	7.5	2	1.3	0	0.0	0	0.0	0	0.0	1	0.6
Beef, patty	90	57.0	20	12.7	36	22.8	7	4.4	2	1.3	1	0.6	1	0.6	0	0.0	1	0.6
Beef sausage	57	35.6	32	20.0	35	21.9	26	16.3	7	4.4	3	1.9	0	0.0	0	0.0	0	0.0
Cheese, cheddar	69	43.1	26	16.3	19	11.9	32	20.0	8	5.0	3	1.9	2	1.3	0	0.0	1	0.6

Table S4. Results of the food frequency analysis continued

	Never/ less than once a month		1-3 times per month		Once a week		2-4 times per week		5-6 times per week		7 times per week		2-3 times per day		4-5 times per day		>6 times per day	
	n	%	n	%	n	%	n	%	n	%	n	%	n	%	n	%	n	%
Chicken, cooked	22	13.7	29	18.0	47	29.2	49	30.4	11	6.8	1	.6	2	1.2	0	0.0	0	0.0
Chicken, stewed	30	18.9	22	13.8	44	27.7	40	25.2	14	8.8	6	3.8	2	1.3	1	0.6	0	0.0
Chicken, feet	38	23.9	44	27.7	28	17.6	37	23.3	6	3.8	2	1.3	3	1.9	1	0.6	0	0.0
Eggs	48	30.4	21	13.3	16	10.1	35	22.2	22	13.9	10	6.3	4	2.5	1	0.6	1	0.6
Fish, hake	99	62.7	28	17.7	15	9.5	10	6.3	4	2.5	1	0.6	1	0.6	0	0.0	0	0.0
Fish, tuna	114	73.1	12	7.7	11	7.1	13	8.3	4	2.6	2	1.3	0	0.0	0	0.0	0	0.0
Lentils	134	84.3	13	8.2	5	3.1	3	1.9	2	1.3	2	1.3	0	0.0	0	0.0	0	0.0
Organ meat (liver, kidney, heart)	48	29.8	65	40.4	27	16.8	13	8.1	4	2.5	2	1.2	0	0.0	2	1.2%	0	.0%
Peanut butter	81	50.3	21	13.0	22	13.7	22	13.7	4	2.5	7	4.3	2	1.2	0	0.0	2	1.2
Pilchards, canned	60	37.3	27	16.8	36	22.4	28	17.4	6	3.7	1	0.6	2	1.2	1	0.6	0	0.0
Polony	45	28.3	30	18.9	29	18.2	22	13.8	22	13.8	10	6.3	1	0.6	0	0.0	0	0.0
Pork, bacon	112	70.0	20	12.5	13	8.1	10	6.3	2	1.3	1	0.6	2	1.3	0	0.0	0	0.0
Pork, ham	121	76.6	16	10.1	7	4.4	5	3.2	7	4.4	2	1.3	0	0.0	0	0.0	0	0.0
Pork, sausages	126	78.3	18	11.2	7	4.3	6	3.7	2	1.2	1	0.6	0	0.0	0	0.0	1	0.6
Viennas	79	49.7	34	21.4	23	14.5	16	10.1	4	2.5	3	1.9	0	0.0	0	0.0	0	0.0
**Fats**																		
Avocado, medium	72	45.9	33	21.0	15	9.6	20	12.7	7	4.5	7	4.5	3	1.9	0	0.0	0	0.0
Holsum	110	69.2	19	11.9	10	6.3	9	5.7	8	5.0	3	1.9	0	0.0	0	0.0	0	0.0
Margarine, brick	83	51.9	19	11.9	6	3.8	15	9.4	18	11.3	12	7.5	6	3.8	1	0.6	0	0.0
Margarine, tub	89	56.0	22	13.8	7	4.4	21	13.2	12	7.5	5	3.1	2	1.3	0	0.0	1	0.6
Mayonnaise	44	27.7	22	13.8	20	12.6	31	19.5	24	15.1	6	3.8	7	4.4	1	0.6	4	2.5
Oil	37	23.7	20	12.8	4	2.6	10	6.4	45	28.8	14	9.0	15	9.6	3	1.9	7	4.5
**Other carbohydrates**																		
Cake, icing	90	55.9	42	26.1	13	8.1	7	4.3	4	2.5	4	2.5	0	0.0	0	0.0	1	0.6
Cake, plain	69	43.1	52	32.5	15	9.4	10	6.3	6	3.8	2	1.3	4	2.5	0	0.0	2	1.3
Chocolate	76	47.5	46	28.8	22	13.8	11	6.9	1	0.6	3	1.9	0	0.0	1	0.6	0	0.0
Cupcake	95	59.7	35	22.0	15	9.4	9	5.7	3	1.9	2	1.3	0	0.0	0	0.0	0	0.0

Table S4. Results of the food frequency analysis continued

	Never/ less than once a month		1-3 times per month		Once a week		2-4 times per week		5-6 times per week		7 times per week		2-3 times per day		4-5 times per day		>6 times per day	
	**n**	%	**n**	%	**n**	%	**n**	%	**n**	%	**n**	%	**n**	%	**n**	%	**n**	%
Chips (Simba)	45	28.8	40	25.6	25	16.0	29	18.6	8	5.1	6	3.8	3	1.9	0	0.0	0	0.0
Sweets	33	21.9	28	18.5	21	13.9	30	19.9	21	13.9	5	3.3	7	4.6	4	2.6	2	1.3

Table S5. The mean frequency scores for commonly consumed food items

Food items	n[2]	Mean[3]	Food items	n	Mean	Food items	n	Mean
Oil	156	3.37	*Mageu*[4]	159	1.82	Beef, sausage	160	1.39
Onions	159	3.31	Orange	156	1.79	Samp and beans	160	1.37
Phutu[5]	158	2.63	*Maas*[6]	156	1.74	Steamed bread (*Ujeqe*)	99	1.34
Sweets	151	2.35	Fruit juice	159	1.73	Peanut butter	161	1.30
Brown bread/roll	145	2.33	Chicken, feet	159	1.70	Avocado, medium	157	1.30
Tomato	157	2.29	Cabbage	161	1.68	Grapes, small	157	1.27
Mayonnaise	159	2.28	Potato crisps	156	1.65	Organ meat (liver, kidney, heart)	161	1.23
Rice, white	156	2.15	Mealie/corn (on cob)	156	1.63	*Isijingi*[7]	24	1.21
Apple, unpeeled medium	159	2.13	Baked beans	160	1.61	Margarine, tub	159	1.21
Eggs	158	2.12	Sweet potato	157	1.62	Instant noodles	158	1.19
Chicken, stewed	159	2.11	Squash, butternut, pumpkin, winter squash	159	1.59	Scone	159	1.18
Chicken, cooked	161	2.06	*Amadumbe*[8]	159	1.58	*Imifino* (Indigenous green leafy vegetables)	23	1.17
Pepper	159	2.01	Beef, cuts	161	1.58	Cake, plain	160	1.16
White bread/roll	157	1.99	Margarine, brick	160	1.57	Biscuit	160	1.14
Maize meal porridge	158	1.93	Cornflakes	160	1.56	Ice cream	158	1.09
Full cream milk	160	1.91	Weetbix	155	1.54	Custard Ultra Mel	159	1.09
Potato, pumpkin	159	1.90	Fried chips	158	1.50	Pear	156	1.09
Mixed vegetable	160	1.88	Vetkoek (*amagwinya*)[9]	144	1.47	Low fat milk	160	1.07
Polony	159	1.87	Pilchards, canned	161	1.43	Beetroot	156	1.06
Banana, small	158	1.84	Cheese, cheddar	160	1.42	Canned fruit	159	1.03
Spinach	155	1.82	Maize meal, stiff	157	1.41	Biscuit, filling	150	1.01

[2] n: Indicates the number of households that consumed that particular food item.
[3] The mean is expressed as an average score.
[4] *Mageu*: fermented maize meal.
[5] *Phutu*: maize meal cooked into a crumbly porridge.
[6] *Maas*: fermented milk product.
[7] *Isijingi*: soft porridge made with pumpkin.
[8] *Amadumbe*: a tuber that grows underground.
[9] *Amagwinya*: traditional fried bread made with flour, yeast, sugar and salt.

Table S5. The mean frequency scores for commonly consumed food items continued

Food items	n	Mean	Food items	n	Mean	Food items	n	Mean
Viennas	159	1.00	Beans, green	158	0.77	Future life	143	0.55
Rice, brown	158	0.99	Fish, hake	158	0.73	Guava	11	0.55
Pasta	160	0.98	Cupcake	159	0.72	Pork, ham	158	0.53
Yoghurt, flavoured	157	0.94	Popcorn with oil	158	0.72	Yoghurt low fat	158	0.46
Fruit salad, fresh	159	0.94	Holsum	159	0.71	Pork sausage	161	0.44
Chocolate	160	0.93	Custard, sweetened full cream	157	0.68	Morvite	12	0.42
Beef, patty	158	0.89	Cucumber	160	0.65	Oats	157	0.38
Lettuce	160	0.88	Maltabella	157	0.64	Pronutro	156	0.37
Beans, lentils and peas (cooked)	160	0.88	Fish, tuna	156	0.63	Cauliflower	160	0.36
Beef mince	159	0.87	Pork, bacon	160	0.63	Yoghurt plain	158	0.35
Peas, green	157	0.86	Mushroom	158	0.61	Lentils	159	0.31
Bean salad, no oil	159	0.85	Cream crackers	160	0.61			
Cake, with icing	161	0.83	Broccoli	159	0.58			
